# A search for pair-produced resonances in four-jet final states at $$\sqrt{s}=13$$$$\text {TeV}$$ with the ATLAS detector

**DOI:** 10.1140/epjc/s10052-018-5693-4

**Published:** 2018-03-22

**Authors:** M. Aaboud, G. Aad, B. Abbott, O. Abdinov, B. Abeloos, S. H. Abidi, O. S. AbouZeid, N. L. Abraham, H. Abramowicz, H. Abreu, R. Abreu, Y. Abulaiti, B. S. Acharya, S. Adachi, L. Adamczyk, J. Adelman, M. Adersberger, T. Adye, A. A. Affolder, T. Agatonovic-Jovin, C. Agheorghiesei, J. A. Aguilar-Saavedra, S. P. Ahlen, F. Ahmadov, G. Aielli, S. Akatsuka, H. Akerstedt, T. P. A. Åkesson, E. Akilli, A. V. Akimov, G. L. Alberghi, J. Albert, P. Albicocco, M. J. Alconada Verzini, S. C. Alderweireldt, M. Aleksa, I. N. Aleksandrov, C. Alexa, G. Alexander, T. Alexopoulos, M. Alhroob, B. Ali, M. Aliev, G. Alimonti, J. Alison, S. P. Alkire, B. M. M. Allbrooke, B. W. Allen, P. P. Allport, A. Aloisio, A. Alonso, F. Alonso, C. Alpigiani, A. A. Alshehri, M. I. Alstaty, B. Alvarez Gonzalez, D. Álvarez Piqueras, M. G. Alviggi, B. T. Amadio, Y. Amaral Coutinho, C. Amelung, D. Amidei, S. P. Amor Dos Santos, A. Amorim, S. Amoroso, G. Amundsen, C. Anastopoulos, L. S. Ancu, N. Andari, T. Andeen, C. F. Anders, J. K. Anders, K. J. Anderson, A. Andreazza, V. Andrei, S. Angelidakis, I. Angelozzi, A. Angerami, A. V. Anisenkov, N. Anjos, A. Annovi, C. Antel, M. Antonelli, A. Antonov, D. J. Antrim, F. Anulli, M. Aoki, L. Aperio Bella, G. Arabidze, Y. Arai, J. P. Araque, V. Araujo Ferraz, A. T. H. Arce, R. E. Ardell, F. A. Arduh, J-F. Arguin, S. Argyropoulos, M. Arik, A. J. Armbruster, L. J. Armitage, O. Arnaez, H. Arnold, M. Arratia, O. Arslan, A. Artamonov, G. Artoni, S. Artz, S. Asai, N. Asbah, A. Ashkenazi, L. Asquith, K. Assamagan, R. Astalos, M. Atkinson, N. B. Atlay, K. Augsten, G. Avolio, B. Axen, M. K. Ayoub, G. Azuelos, A. E. Baas, M. J. Baca, H. Bachacou, K. Bachas, M. Backes, M. Backhaus, P. Bagnaia, M. Bahmani, H. Bahrasemani, J. T. Baines, M. Bajic, O. K. Baker, E. M. Baldin, P. Balek, F. Balli, W. K. Balunas, E. Banas, A. Bandyopadhyay, Sw. Banerjee, A. A. E. Bannoura, L. Barak, E. L. Barberio, D. Barberis, M. Barbero, T. Barillari, M-S Barisits, J. T. Barkeloo, T. Barklow, N. Barlow, S. L. Barnes, B. M. Barnett, R. M. Barnett, Z. Barnovska-Blenessy, A. Baroncelli, G. Barone, A. J. Barr, L. Barranco Navarro, F. Barreiro, J. Barreiro Guimarães da Costa, R. Bartoldus, A. E. Barton, P. Bartos, A. Basalaev, A. Bassalat, R. L. Bates, S. J. Batista, J. R. Batley, M. Battaglia, M. Bauce, F. Bauer, H. S. Bawa, J. B. Beacham, M. D. Beattie, T. Beau, P. H. Beauchemin, P. Bechtle, H. P. Beck, H. C. Beck, K. Becker, M. Becker, M. Beckingham, C. Becot, A. J. Beddall, A. Beddall, V. A. Bednyakov, M. Bedognetti, C. P. Bee, T. A. Beermann, M. Begalli, M. Begel, J. K. Behr, A. S. Bell, G. Bella, L. Bellagamba, A. Bellerive, M. Bellomo, K. Belotskiy, O. Beltramello, N. L. Belyaev, O. Benary, D. Benchekroun, M. Bender, K. Bendtz, N. Benekos, Y. Benhammou, E. Benhar Noccioli, J. Benitez, D. P. Benjamin, M. Benoit, J. R. Bensinger, S. Bentvelsen, L. Beresford, M. Beretta, D. Berge, E. Bergeaas Kuutmann, N. Berger, J. Beringer, S. Berlendis, N. R. Bernard, G. Bernardi, C. Bernius, F. U. Bernlochner, T. Berry, P. Berta, C. Bertella, G. Bertoli, F. Bertolucci, I. A. Bertram, C. Bertsche, D. Bertsche, G. J. Besjes, O. Bessidskaia Bylund, M. Bessner, N. Besson, C. Betancourt, A. Bethani, S. Bethke, A. J. Bevan, J. Beyer, R. M. Bianchi, O. Biebel, D. Biedermann, R. Bielski, K. Bierwagen, N. V. Biesuz, M. Biglietti, T. R. V. Billoud, H. Bilokon, M. Bindi, A. Bingul, C. Bini, S. Biondi, T. Bisanz, C. Bittrich, D. M. Bjergaard, C. W. Black, J. E. Black, K. M. Black, R. E. Blair, T. Blazek, I. Bloch, C. Blocker, A. Blue, W. Blum, U. Blumenschein, S. Blunier, G. J. Bobbink, V. S. Bobrovnikov, S. S. Bocchetta, A. Bocci, C. Bock, M. Boehler, D. Boerner, D. Bogavac, A. G. Bogdanchikov, C. Bohm, V. Boisvert, P. Bokan, T. Bold, A. S. Boldyrev, A. E. Bolz, M. Bomben, M. Bona, M. Boonekamp, A. Borisov, G. Borissov, J. Bortfeldt, D. Bortoletto, V. Bortolotto, D. Boscherini, M. Bosman, J. D. Bossio Sola, J. Boudreau, J. Bouffard, E. V. Bouhova-Thacker, D. Boumediene, C. Bourdarios, S. K. Boutle, A. Boveia, J. Boyd, I. R. Boyko, J. Bracinik, A. Brandt, G. Brandt, O. Brandt, U. Bratzler, B. Brau, J. E. Brau, W. D. Breaden Madden, K. Brendlinger, A. J. Brennan, L. Brenner, R. Brenner, S. Bressler, D. L. Briglin, T. M. Bristow, D. Britton, D. Britzger, F. M. Brochu, I. Brock, R. Brock, G. Brooijmans, T. Brooks, W. K. Brooks, J. Brosamer, E. Brost, J. H Broughton, P. A. Bruckman de Renstrom, D. Bruncko, A. Bruni, G. Bruni, L. S. Bruni, BH Brunt, M. Bruschi, N. Bruscino, P. Bryant, L. Bryngemark, T. Buanes, Q. Buat, P. Buchholz, A. G. Buckley, I. A. Budagov, F. Buehrer, M. K. Bugge, O. Bulekov, D. Bullock, T. J. Burch, S. Burdin, C. D. Burgard, A. M. Burger, B. Burghgrave, K. Burka, S. Burke, I. Burmeister, J. T. P. Burr, E. Busato, D. Büscher, V. Büscher, P. Bussey, J. M. Butler, C. M. Buttar, J. M. Butterworth, P. Butti, W. Buttinger, A. Buzatu, A. R. Buzykaev, S. Cabrera Urbán, D. Caforio, V. M. Cairo, O. Cakir, N. Calace, P. Calafiura, A. Calandri, G. Calderini, P. Calfayan, G. Callea, L. P. Caloba, S. Calvente Lopez, D. Calvet, S. Calvet, T. P. Calvet, R. Camacho Toro, S. Camarda, P. Camarri, D. Cameron, R. Caminal Armadans, C. Camincher, S. Campana, M. Campanelli, A. Camplani, A. Campoverde, V. Canale, M. Cano Bret, J. Cantero, T. Cao, M. D. M. Capeans Garrido, I. Caprini, M. Caprini, M. Capua, R. M. Carbone, R. Cardarelli, F. Cardillo, I. Carli, T. Carli, G. Carlino, B. T. Carlson, L. Carminati, R. M. D. Carney, S. Caron, E. Carquin, S. Carrá, G. D. Carrillo-Montoya, J. Carvalho, D. Casadei, M. P. Casado, M. Casolino, D. W. Casper, R. Castelijn, V. Castillo Gimenez, N. F. Castro, A. Catinaccio, J. R. Catmore, A. Cattai, J. Caudron, V. Cavaliere, E. Cavallaro, D. Cavalli, M. Cavalli-Sforza, V. Cavasinni, E. Celebi, F. Ceradini, L. Cerda Alberich, A. S. Cerqueira, A. Cerri, L. Cerrito, F. Cerutti, A. Cervelli, S. A. Cetin, A. Chafaq, D. Chakraborty, S. K. Chan, W. S. Chan, Y. L. Chan, P. Chang, J. D. Chapman, D. G. Charlton, C. C. Chau, C. A. Chavez Barajas, S. Che, S. Cheatham, A. Chegwidden, S. Chekanov, S. V. Chekulaev, G. A. Chelkov, M. A. Chelstowska, C. Chen, H. Chen, J. Chen, S. Chen, S. Chen, X. Chen, Y. Chen, H. C. Cheng, H. J. Cheng, A. Cheplakov, E. Cheremushkina, R. Cherkaoui El Moursli, E. Cheu, K. Cheung, L. Chevalier, V. Chiarella, G. Chiarelli, G. Chiodini, A. S. Chisholm, A. Chitan, Y. H. Chiu, M. V. Chizhov, K. Choi, A. R. Chomont, S. Chouridou, V. Christodoulou, D. Chromek-Burckhart, M. C. Chu, J. Chudoba, A. J. Chuinard, J. J. Chwastowski, L. Chytka, A. K. Ciftci, D. Cinca, V. Cindro, I. A. Cioara, C. Ciocca, A. Ciocio, F. Cirotto, Z. H. Citron, M. Citterio, M. Ciubancan, A. Clark, B. L. Clark, M. R. Clark, P. J. Clark, R. N. Clarke, C. Clement, Y. Coadou, M. Cobal, A. Coccaro, J. Cochran, L. Colasurdo, B. Cole, A. P. Colijn, J. Collot, T. Colombo, P. Conde Muiño, E. Coniavitis, S. H. Connell, I. A. Connelly, S. Constantinescu, G. Conti, F. Conventi, M. Cooke, A. M. Cooper-Sarkar, F. Cormier, K. J. R. Cormier, M. Corradi, F. Corriveau, A. Cortes-Gonzalez, G. Cortiana, G. Costa, M. J. Costa, D. Costanzo, G. Cottin, G. Cowan, B. E. Cox, K. Cranmer, S. J. Crawley, R. A. Creager, G. Cree, S. Crépé-Renaudin, F. Crescioli, W. A. Cribbs, M. Cristinziani, V. Croft, G. Crosetti, A. Cueto, T. Cuhadar Donszelmann, A. R. Cukierman, J. Cummings, M. Curatolo, J. Cúth, S. Czekierda, P. Czodrowski, G. D’amen, S. D’Auria, L. D’eramo, M. D’Onofrio, M. J. Da Cunha Sargedas De Sousa, C. Da Via, W. Dabrowski, T. Dado, T. Dai, O. Dale, F. Dallaire, C. Dallapiccola, M. Dam, J. R. Dandoy, M. F. Daneri, N. P. Dang, A. C. Daniells, N. S. Dann, M. Danninger, M. Dano Hoffmann, V. Dao, G. Darbo, S. Darmora, J. Dassoulas, A. Dattagupta, T. Daubney, W. Davey, C. David, T. Davidek, D. R. Davis, P. Davison, E. Dawe, I. Dawson, K. De, R. de Asmundis, A. De Benedetti, S. De Castro, S. De Cecco, N. De Groot, P. de Jong, H. De la Torre, F. De Lorenzi, A. De Maria, D. De Pedis, A. De Salvo, U. De Sanctis, A. De Santo, K. De Vasconcelos Corga, J. B. De Vivie De Regie, W. J. Dearnaley, R. Debbe, C. Debenedetti, D. V. Dedovich, N. Dehghanian, I. Deigaard, M. Del Gaudio, J. Del Peso, D. Delgove, F. Deliot, C. M. Delitzsch, A. Dell’Acqua, L. Dell’Asta, M. Dell’Orso, M. Della Pietra, D. della Volpe, M. Delmastro, C. Delporte, P. A. Delsart, D. A. DeMarco, S. Demers, M. Demichev, A. Demilly, S. P. Denisov, D. Denysiuk, D. Derendarz, J. E. Derkaoui, F. Derue, P. Dervan, K. Desch, C. Deterre, K. Dette, M. R. Devesa, P. O. Deviveiros, A. Dewhurst, S. Dhaliwal, F. A. Di Bello, A. Di Ciaccio, L. Di Ciaccio, W. K. Di Clemente, C. Di Donato, A. Di Girolamo, B. Di Girolamo, B. Di Micco, R. Di Nardo, K. F. Di Petrillo, A. Di Simone, R. Di Sipio, D. Di Valentino, C. Diaconu, M. Diamond, F. A. Dias, M. A. Diaz, E. B. Diehl, J. Dietrich, S. Díez Cornell, A. Dimitrievska, J. Dingfelder, P. Dita, S. Dita, F. Dittus, F. Djama, T. Djobava, J. I. Djuvsland, M. A. B. do Vale, D. Dobos, M. Dobre, C. Doglioni, J. Dolejsi, Z. Dolezal, M. Donadelli, S. Donati, P. Dondero, J. Donini, J. Dopke, A. Doria, M. T. Dova, A. T. Doyle, E. Drechsler, M. Dris, Y. Du, J. Duarte-Campderros, A. Dubreuil, E. Duchovni, G. Duckeck, A. Ducourthial, O. A. Ducu, D. Duda, A. Dudarev, A. Chr. Dudder, E. M. Duffield, L. Duflot, M. Dührssen, M. Dumancic, A. E. Dumitriu, A. K. Duncan, M. Dunford, H. Duran Yildiz, M. Düren, A. Durglishvili, D. Duschinger, B. Dutta, D. Duvnjak, M. Dyndal, B. S. Dziedzic, C. Eckardt, K. M. Ecker, R. C. Edgar, T. Eifert, G. Eigen, K. Einsweiler, T. Ekelof, M. El Kacimi, R. El Kosseifi, V. Ellajosyula, M. Ellert, S. Elles, F. Ellinghaus, A. A. Elliot, N. Ellis, J. Elmsheuser, M. Elsing, D. Emeliyanov, Y. Enari, O. C. Endner, J. S. Ennis, J. Erdmann, A. Ereditato, M. Ernst, S. Errede, M. Escalier, C. Escobar, B. Esposito, O. Estrada Pastor, A. I. Etienvre, E. Etzion, H. Evans, A. Ezhilov, M. Ezzi, F. Fabbri, L. Fabbri, V. Fabiani, G. Facini, R. M. Fakhrutdinov, S. Falciano, R. J. Falla, J. Faltova, Y. Fang, M. Fanti, A. Farbin, A. Farilla, C. Farina, E. M. Farina, T. Farooque, S. Farrell, S. M. Farrington, P. Farthouat, F. Fassi, P. Fassnacht, D. Fassouliotis, M. Faucci Giannelli, A. Favareto, W. J. Fawcett, L. Fayard, O. L. Fedin, W. Fedorko, S. Feigl, L. Feligioni, C. Feng, E. J. Feng, H. Feng, M. J. Fenton, A. B. Fenyuk, L. Feremenga, P. Fernandez Martinez, S. Fernandez Perez, J. Ferrando, A. Ferrari, P. Ferrari, R. Ferrari, D. E. Ferreira de Lima, A. Ferrer, D. Ferrere, C. Ferretti, F. Fiedler, A. Filipčič, M. Filipuzzi, F. Filthaut, M. Fincke-Keeler, K. D. Finelli, M. C. N. Fiolhais, L. Fiorini, A. Fischer, C. Fischer, J. Fischer, W. C. Fisher, N. Flaschel, I. Fleck, P. Fleischmann, R. R. M. Fletcher, T. Flick, B. M. Flierl, L. R. Flores Castillo, M. J. Flowerdew, G. T. Forcolin, A. Formica, F. A. Förster, A. Forti, A. G. Foster, D. Fournier, H. Fox, S. Fracchia, P. Francavilla, M. Franchini, S. Franchino, D. Francis, L. Franconi, M. Franklin, M. Frate, M. Fraternali, D. Freeborn, S. M. Fressard-Batraneanu, B. Freund, D. Froidevaux, J. A. Frost, C. Fukunaga, T. Fusayasu, J. Fuster, C. Gabaldon, O. Gabizon, A. Gabrielli, A. Gabrielli, G. P. Gach, S. Gadatsch, S. Gadomski, G. Gagliardi, L. G. Gagnon, C. Galea, B. Galhardo, E. J. Gallas, B. J. Gallop, P. Gallus, G. Galster, K. K. Gan, S. Ganguly, Y. Gao, Y. S. Gao, F. M. Garay Walls, C. García, J. E. García Navarro, J. A. García Pascual, M. Garcia-Sciveres, R. W. Gardner, N. Garelli, V. Garonne, A. Gascon Bravo, K. Gasnikova, C. Gatti, A. Gaudiello, G. Gaudio, I. L. Gavrilenko, C. Gay, G. Gaycken, E. N. Gazis, C. N. P. Gee, J. Geisen, M. Geisen, M. P. Geisler, K. Gellerstedt, C. Gemme, M. H. Genest, C. Geng, S. Gentile, C. Gentsos, S. George, D. Gerbaudo, A. Gershon, G. Geßner, S. Ghasemi, M. Ghneimat, B. Giacobbe, S. Giagu, N. Giangiacomi, P. Giannetti, S. M. Gibson, M. Gignac, M. Gilchriese, D. Gillberg, G. Gilles, D. M. Gingrich, N. Giokaris, M. P. Giordani, F. M. Giorgi, P. F. Giraud, P. Giromini, G. Giugliarelli, D. Giugni, F. Giuli, C. Giuliani, M. Giulini, B. K. Gjelsten, S. Gkaitatzis, I. Gkialas, E. L. Gkougkousis, P. Gkountoumis, L. K. Gladilin, C. Glasman, J. Glatzer, P. C. F. Glaysher, A. Glazov, M. Goblirsch-Kolb, J. Godlewski, S. Goldfarb, T. Golling, D. Golubkov, A. Gomes, R. Gonçalo, R. Goncalves Gama, J. Goncalves Pinto Firmino Da Costa, G. Gonella, L. Gonella, A. Gongadze, S. González de la Hoz, S. Gonzalez-Sevilla, L. Goossens, P. A. Gorbounov, H. A. Gordon, I. Gorelov, B. Gorini, E. Gorini, A. Gorišek, A. T. Goshaw, C. Gössling, M. I. Gostkin, C. A. Gottardo, C. R. Goudet, D. Goujdami, A. G. Goussiou, N. Govender, E. Gozani, L. Graber, I. Grabowska-Bold, P. O. J. Gradin, J. Gramling, E. Gramstad, S. Grancagnolo, V. Gratchev, P. M. Gravila, C. Gray, H. M. Gray, Z. D. Greenwood, C. Grefe, K. Gregersen, I. M. Gregor, P. Grenier, K. Grevtsov, J. Griffiths, A. A. Grillo, K. Grimm, S. Grinstein, Ph. Gris, J.-F. Grivaz, S. Groh, E. Gross, J. Grosse-Knetter, G. C. Grossi, Z. J. Grout, A. Grummer, L. Guan, W. Guan, J. Guenther, F. Guescini, D. Guest, O. Gueta, B. Gui, E. Guido, T. Guillemin, S. Guindon, U. Gul, C. Gumpert, J. Guo, W. Guo, Y. Guo, R. Gupta, S. Gupta, G. Gustavino, P. Gutierrez, N. G. Gutierrez Ortiz, C. Gutschow, C. Guyot, M. P. Guzik, C. Gwenlan, C. B. Gwilliam, A. Haas, C. Haber, H. K. Hadavand, N. Haddad, A. Hadef, S. Hageböck, M. Hagihara, H. Hakobyan, M. Haleem, J. Haley, G. Halladjian, G. D. Hallewell, K. Hamacher, P. Hamal, K. Hamano, A. Hamilton, G. N. Hamity, P. G. Hamnett, L. Han, S. Han, K. Hanagaki, K. Hanawa, M. Hance, B. Haney, P. Hanke, J. B. Hansen, J. D. Hansen, M. C. Hansen, P. H. Hansen, K. Hara, A. S. Hard, T. Harenberg, F. Hariri, S. Harkusha, R. D. Harrington, P. F. Harrison, N. M. Hartmann, M. Hasegawa, Y. Hasegawa, A. Hasib, S. Hassani, S. Haug, R. Hauser, L. Hauswald, L. B. Havener, M. Havranek, C. M. Hawkes, R. J. Hawkings, D. Hayakawa, D. Hayden, C. P. Hays, J. M. Hays, H. S. Hayward, S. J. Haywood, S. J. Head, T. Heck, V. Hedberg, L. Heelan, S. Heer, K. K. Heidegger, S. Heim, T. Heim, B. Heinemann, J. J. Heinrich, L. Heinrich, C. Heinz, J. Hejbal, L. Helary, A. Held, S. Hellman, C. Helsens, R. C. W. Henderson, Y. Heng, S. Henkelmann, A. M. Henriques Correia, S. Henrot-Versille, G. H. Herbert, H. Herde, V. Herget, Y. Hernández Jiménez, H. Herr, G. Herten, R. Hertenberger, L. Hervas, T. C. Herwig, G. G. Hesketh, N. P. Hessey, J. W. Hetherly, S. Higashino, E. Higón-Rodriguez, K. Hildebrand, E. Hill, J. C. Hill, K. H. Hiller, S. J. Hillier, M. Hils, I. Hinchliffe, M. Hirose, D. Hirschbuehl, B. Hiti, O. Hladik, X. Hoad, J. Hobbs, N. Hod, M. C. Hodgkinson, P. Hodgson, A. Hoecker, M. R. Hoeferkamp, F. Hoenig, D. Hohn, T. R. Holmes, M. Homann, S. Honda, T. Honda, T. M. Hong, B. H. Hooberman, W. H. Hopkins, Y. Horii, A. J. Horton, J-Y. Hostachy, S. Hou, A. Hoummada, J. Howarth, J. Hoya, M. Hrabovsky, J. Hrdinka, I. Hristova, J. Hrivnac, T. Hryn’ova, A. Hrynevich, P. J. Hsu, S.-C. Hsu, Q. Hu, S. Hu, Y. Huang, Z. Hubacek, F. Hubaut, F. Huegging, T. B. Huffman, E. W. Hughes, G. Hughes, M. Huhtinen, P. Huo, N. Huseynov, J. Huston, J. Huth, G. Iacobucci, G. Iakovidis, I. Ibragimov, L. Iconomidou-Fayard, Z. Idrissi, P. Iengo, O. Igonkina, T. Iizawa, Y. Ikegami, M. Ikeno, Y. Ilchenko, D. Iliadis, N. Ilic, G. Introzzi, P. Ioannou, M. Iodice, K. Iordanidou, V. Ippolito, M. F. Isacson, N. Ishijima, M. Ishino, M. Ishitsuka, C. Issever, S. Istin, F. Ito, J. M. Iturbe Ponce, R. Iuppa, H. Iwasaki, J. M. Izen, V. Izzo, S. Jabbar, P. Jackson, R. M. Jacobs, V. Jain, K. B. Jakobi, K. Jakobs, S. Jakobsen, T. Jakoubek, D. O. Jamin, D. K. Jana, R. Jansky, J. Janssen, M. Janus, P. A. Janus, G. Jarlskog, N. Javadov, T. Javůrek, M. Javurkova, F. Jeanneau, L. Jeanty, J. Jejelava, A. Jelinskas, P. Jenni, C. Jeske, S. Jézéquel, H. Ji, J. Jia, H. Jiang, Y. Jiang, Z. Jiang, S. Jiggins, J. Jimenez Pena, S. Jin, A. Jinaru, O. Jinnouchi, H. Jivan, P. Johansson, K. A. Johns, C. A. Johnson, W. J. Johnson, K. Jon-And, R. W. L. Jones, S. D. Jones, S. Jones, T. J. Jones, J. Jongmanns, P. M. Jorge, J. Jovicevic, X. Ju, A. Juste Rozas, M. K. Köhler, A. Kaczmarska, M. Kado, H. Kagan, M. Kagan, S. J. Kahn, T. Kaji, E. Kajomovitz, C. W. Kalderon, A. Kaluza, S. Kama, A. Kamenshchikov, N. Kanaya, L. Kanjir, V. A. Kantserov, J. Kanzaki, B. Kaplan, L. S. Kaplan, D. Kar, K. Karakostas, N. Karastathis, M. J. Kareem, E. Karentzos, S. N. Karpov, Z. M. Karpova, K. Karthik, V. Kartvelishvili, A. N. Karyukhin, K. Kasahara, L. Kashif, R. D. Kass, A. Kastanas, Y. Kataoka, C. Kato, A. Katre, J. Katzy, K. Kawade, K. Kawagoe, T. Kawamoto, G. Kawamura, E. F. Kay, V. F. Kazanin, R. Keeler, R. Kehoe, J. S. Keller, J. J. Kempster, J Kendrick, H. Keoshkerian, O. Kepka, B. P. Kerševan, S. Kersten, R. A. Keyes, M. Khader, F. Khalil-zada, A. Khanov, A. G. Kharlamov, T. Kharlamova, A. Khodinov, T. J. Khoo, V. Khovanskiy, E. Khramov, J. Khubua, S. Kido, C. R. Kilby, H. Y. Kim, S. H. Kim, Y. K. Kim, N. Kimura, O. M. Kind, B. T. King, D. Kirchmeier, J. Kirk, A. E. Kiryunin, T. Kishimoto, D. Kisielewska, V. Kitali, K. Kiuchi, O. Kivernyk, E. Kladiva, T. Klapdor-Kleingrothaus, M. H. Klein, M. Klein, U. Klein, K. Kleinknecht, P. Klimek, A. Klimentov, R. Klingenberg, T. Klingl, T. Klioutchnikova, E.-E. Kluge, P. Kluit, S. Kluth, E. Kneringer, E. B. F. G. Knoops, A. Knue, A. Kobayashi, D. Kobayashi, T. Kobayashi, M. Kobel, M. Kocian, P. Kodys, T. Koffas, E. Koffeman, N. M. Köhler, T. Koi, M. Kolb, I. Koletsou, A. A. Komar, Y. Komori, T. Kondo, N. Kondrashova, K. Köneke, A. C. König, T. Kono, R. Konoplich, N. Konstantinidis, R. Kopeliansky, S. Koperny, A. K. Kopp, K. Korcyl, K. Kordas, A. Korn, A. A. Korol, I. Korolkov, E. V. Korolkova, O. Kortner, S. Kortner, T. Kosek, V. V. Kostyukhin, A. Kotwal, A. Koulouris, A. Kourkoumeli-Charalampidi, C. Kourkoumelis, E. Kourlitis, V. Kouskoura, A. B. Kowalewska, R. Kowalewski, T. Z. Kowalski, C. Kozakai, W. Kozanecki, A. S. Kozhin, V. A. Kramarenko, G. Kramberger, D. Krasnopevtsev, M. W. Krasny, A. Krasznahorkay, D. Krauss, J. A. Kremer, J. Kretzschmar, K. Kreutzfeldt, P. Krieger, K. Krizka, K. Kroeninger, H. Kroha, J. Kroll, J. Kroll, J. Kroseberg, J. Krstic, U. Kruchonak, H. Krüger, N. Krumnack, M. C. Kruse, T. Kubota, H. Kucuk, S. Kuday, J. T. Kuechler, S. Kuehn, A. Kugel, F. Kuger, T. Kuhl, V. Kukhtin, R. Kukla, Y. Kulchitsky, S. Kuleshov, Y. P. Kulinich, M. Kuna, T. Kunigo, A. Kupco, T. Kupfer, O. Kuprash, H. Kurashige, L. L. Kurchaninov, Y. A. Kurochkin, M. G. Kurth, V. Kus, E. S. Kuwertz, M. Kuze, J. Kvita, T. Kwan, D. Kyriazopoulos, A. La Rosa, J. L. La Rosa Navarro, L. La Rotonda, F. La Ruffa, C. Lacasta, F. Lacava, J. Lacey, H. Lacker, D. Lacour, E. Ladygin, R. Lafaye, B. Laforge, T. Lagouri, S. Lai, S. Lammers, W. Lampl, E. Lançon, U. Landgraf, M. P. J. Landon, M. C. Lanfermann, V. S. Lang, J. C. Lange, R. J. Langenberg, A. J. Lankford, F. Lanni, K. Lantzsch, A. Lanza, A. Lapertosa, S. Laplace, J. F. Laporte, T. Lari, F. Lasagni Manghi, M. Lassnig, P. Laurelli, W. Lavrijsen, A. T. Law, P. Laycock, T. Lazovich, M. Lazzaroni, B. Le, O. Le Dortz, E. Le Guirriec, E. P. Le Quilleuc, M. LeBlanc, T. LeCompte, F. Ledroit-Guillon, C. A. Lee, G. R. Lee, S. C. Lee, L. Lee, B. Lefebvre, G. Lefebvre, M. Lefebvre, F. Legger, C. Leggett, G. Lehmann Miotto, X. Lei, W. A. Leight, M. A. L. Leite, R. Leitner, D. Lellouch, B. Lemmer, K. J. C. Leney, T. Lenz, B. Lenzi, R. Leone, S. Leone, C. Leonidopoulos, G. Lerner, C. Leroy, A. A. J. Lesage, C. G. Lester, M. Levchenko, J. Levêque, D. Levin, L. J. Levinson, M. Levy, D. Lewis, B. Li, Changqiao Li, H. Li, L. Li, Q. Li, S. Li, X. Li, Y. Li, Z. Liang, B. Liberti, A. Liblong, K. Lie, J. Liebal, W. Liebig, A. Limosani, S. C. Lin, T. H. Lin, R. A. Linck, B. E. Lindquist, A. E. Lionti, E. Lipeles, A. Lipniacka, M. Lisovyi, T. M. Liss, A. Lister, A. M. Litke, B. Liu, H. Liu, H. Liu, J. K. K. Liu, J. Liu, J. B. Liu, K. Liu, L. Liu, M. Liu, Y. L. Liu, Y. Liu, M. Livan, A. Lleres, J. Llorente Merino, S. L. Lloyd, C. Y. Lo, F. Lo Sterzo, E. M. Lobodzinska, P. Loch, F. K. Loebinger, A. Loesle, K. M. Loew, A. Loginov, T. Lohse, K. Lohwasser, M. Lokajicek, B. A. Long, J. D. Long, R. E. Long, L. Longo, K. A. Looper, J. A. Lopez, D. Lopez Mateos, I. Lopez Paz, A. Lopez Solis, J. Lorenz, N. Lorenzo Martinez, M. Losada, P. J. Lösel, X. Lou, A. Lounis, J. Love, P. A. Love, H. Lu, N. Lu, Y. J. Lu, H. J. Lubatti, C. Luci, A. Lucotte, C. Luedtke, F. Luehring, W. Lukas, L. Luminari, O. Lundberg, B. Lund-Jensen, M. S. Lutz, P. M. Luzi, D. Lynn, R. Lysak, E. Lytken, F. Lyu, V. Lyubushkin, H. Ma, L. L. Ma, Y. Ma, G. Maccarrone, A. Macchiolo, C. M. Macdonald, B. Maček, J. Machado Miguens, D. Madaffari, R. Madar, W. F. Mader, A. Madsen, J. Maeda, S. Maeland, T. Maeno, A. S. Maevskiy, V. Magerl, J. Mahlstedt, C. Maiani, C. Maidantchik, A. A. Maier, T. Maier, A. Maio, O. Majersky, S. Majewski, Y. Makida, N. Makovec, B. Malaescu, Pa. Malecki, V. P. Maleev, F. Malek, U. Mallik, D. Malon, C. Malone, S. Maltezos, S. Malyukov, J. Mamuzic, G. Mancini, I. Mandić, J. Maneira, L. Manhaes de Andrade Filho, J. Manjarres Ramos, K. H. Mankinen, A. Mann, A. Manousos, B. Mansoulie, J. D. Mansour, R. Mantifel, M. Mantoani, S. Manzoni, L. Mapelli, G. Marceca, L. March, L. Marchese, G. Marchiori, M. Marcisovsky, M. Marjanovic, D. E. Marley, F. Marroquim, S. P. Marsden, Z. Marshall, M. U. F Martensson, S. Marti-Garcia, C. B. Martin, T. A. Martin, V. J. Martin, B. Martin dit Latour, M. Martinez, V. I. Martinez Outschoorn, S. Martin-Haugh, V. S. Martoiu, A. C. Martyniuk, A. Marzin, L. Masetti, T. Mashimo, R. Mashinistov, J. Masik, A. L. Maslennikov, L. Massa, P. Mastrandrea, A. Mastroberardino, T. Masubuchi, P. Mättig, J. Maurer, S. J. Maxfield, D. A. Maximov, R. Mazini, I. Maznas, S. M. Mazza, N. C. Mc Fadden, G. Mc Goldrick, S. P. Mc Kee, A. McCarn, R. L. McCarthy, T. G. McCarthy, L. I. McClymont, E. F. McDonald, J. A. Mcfayden, G. Mchedlidze, S. J. McMahon, P. C. McNamara, R. A. McPherson, S. Meehan, T. J. Megy, S. Mehlhase, A. Mehta, T. Meideck, K. Meier, B. Meirose, D. Melini, B. R. Mellado Garcia, J. D. Mellenthin, M. Melo, F. Meloni, A. Melzer, S. B. Menary, L. Meng, X. T. Meng, A. Mengarelli, S. Menke, E. Meoni, S. Mergelmeyer, P. Mermod, L. Merola, C. Meroni, F. S. Merritt, A. Messina, J. Metcalfe, A. S. Mete, C. Meyer, J-P. Meyer, J. Meyer, H. Meyer Zu Theenhausen, F. Miano, R. P. Middleton, S. Miglioranzi, L. Mijović, G. Mikenberg, M. Mikestikova, M. Mikuž, M. Milesi, A. Milic, D. W. Miller, C. Mills, A. Milov, D. A. Milstead, A. A. Minaenko, Y. Minami, I. A. Minashvili, A. I. Mincer, B. Mindur, M. Mineev, Y. Minegishi, Y. Ming, L. M. Mir, K. P. Mistry, T. Mitani, J. Mitrevski, V. A. Mitsou, A. Miucci, P. S. Miyagawa, A. Mizukami, J. U. Mjörnmark, T. Mkrtchyan, M. Mlynarikova, T. Moa, K. Mochizuki, P. Mogg, S. Mohapatra, S. Molander, R. Moles-Valls, R. Monden, M. C. Mondragon, K. Mönig, J. Monk, E. Monnier, A. Montalbano, J. Montejo Berlingen, F. Monticelli, S. Monzani, R. W. Moore, N. Morange, D. Moreno, M. Moreno Llácer, P. Morettini, S. Morgenstern, D. Mori, T. Mori, M. Morii, M. Morinaga, V. Morisbak, A. K. Morley, G. Mornacchi, J. D. Morris, L. Morvaj, P. Moschovakos, M. Mosidze, H. J. Moss, J. Moss, K. Motohashi, R. Mount, E. Mountricha, E. J. W. Moyse, S. Muanza, F. Mueller, J. Mueller, R. S. P. Mueller, D. Muenstermann, P. Mullen, G. A. Mullier, F. J. Munoz Sanchez, W. J. Murray, H. Musheghyan, M. Muškinja, A. G. Myagkov, M. Myska, B. P. Nachman, O. Nackenhorst, K. Nagai, R. Nagai, K. Nagano, Y. Nagasaka, K. Nagata, M. Nagel, E. Nagy, A. M. Nairz, Y. Nakahama, K. Nakamura, T. Nakamura, I. Nakano, R. F. Naranjo Garcia, R. Narayan, D. I. Narrias Villar, I. Naryshkin, T. Naumann, G. Navarro, R. Nayyar, H. A. Neal, P. Yu. Nechaeva, T. J. Neep, A. Negri, M. Negrini, S. Nektarijevic, C. Nellist, A. Nelson, M. E. Nelson, S. Nemecek, P. Nemethy, M. Nessi, M. S. Neubauer, M. Neumann, P. R. Newman, T. Y. Ng, T. Nguyen Manh, R. B. Nickerson, R. Nicolaidou, J. Nielsen, V. Nikolaenko, I. Nikolic-Audit, K. Nikolopoulos, J. K. Nilsen, P. Nilsson, Y. Ninomiya, A. Nisati, N. Nishu, R. Nisius, I. Nitsche, T. Nitta, T. Nobe, Y. Noguchi, M. Nomachi, I. Nomidis, M. A. Nomura, T. Nooney, M. Nordberg, N. Norjoharuddeen, O. Novgorodova, M. Nozaki, L. Nozka, K. Ntekas, E. Nurse, F. Nuti, K. O’connor, D. C. O’Neil, A. A. O’Rourke, V. O’Shea, F. G. Oakham, H. Oberlack, T. Obermann, J. Ocariz, A. Ochi, I. Ochoa, J. P. Ochoa-Ricoux, S. Oda, S. Odaka, A. Oh, S. H. Oh, C. C. Ohm, H. Ohman, H. Oide, H. Okawa, Y. Okumura, T. Okuyama, A. Olariu, L. F. Oleiro Seabra, S. A. Olivares Pino, D. Oliveira Damazio, A. Olszewski, J. Olszowska, A. Onofre, K. Onogi, P. U. E. Onyisi, H. Oppen, M. J. Oreglia, Y. Oren, D. Orestano, N. Orlando, R. S. Orr, B. Osculati, R. Ospanov, G. Otero y Garzon, H. Otono, M. Ouchrif, F. Ould-Saada, A. Ouraou, K. P. Oussoren, Q. Ouyang, M. Owen, R. E. Owen, V. E. Ozcan, N. Ozturk, K. Pachal, A. Pacheco Pages, L. Pacheco Rodriguez, C. Padilla Aranda, S. Pagan Griso, M. Paganini, F. Paige, G. Palacino, S. Palazzo, S. Palestini, M. Palka, D. Pallin, E. St. Panagiotopoulou, I. Panagoulias, C. E. Pandini, J. G. Panduro Vazquez, P. Pani, S. Panitkin, D. Pantea, L. Paolozzi, Th. D. Papadopoulou, K. Papageorgiou, A. Paramonov, D. Paredes Hernandez, A. J. Parker, M. A. Parker, K. A. Parker, F. Parodi, J. A. Parsons, U. Parzefall, V. R. Pascuzzi, J. M. Pasner, E. Pasqualucci, S. Passaggio, Fr. Pastore, S. Pataraia, J. R. Pater, T. Pauly, B. Pearson, S. Pedraza Lopez, R. Pedro, S. V. Peleganchuk, O. Penc, C. Peng, H. Peng, J. Penwell, B. S. Peralva, M. M. Perego, D. V. Perepelitsa, F. Peri, L. Perini, H. Pernegger, S. Perrella, R. Peschke, V. D. Peshekhonov, K. Peters, R. F. Y. Peters, B. A. Petersen, T. C. Petersen, E. Petit, A. Petridis, C. Petridou, P. Petroff, E. Petrolo, M. Petrov, F. Petrucci, N. E. Pettersson, A. Peyaud, R. Pezoa, F. H. Phillips, P. W. Phillips, G. Piacquadio, E. Pianori, A. Picazio, E. Piccaro, M. A. Pickering, R. Piegaia, J. E. Pilcher, A. D. Pilkington, A. W. J. Pin, M. Pinamonti, J. L. Pinfold, H. Pirumov, M. Pitt, L. Plazak, M.-A. Pleier, V. Pleskot, E. Plotnikova, D. Pluth, P. Podberezko, R. Poettgen, R. Poggi, L. Poggioli, D. Pohl, G. Polesello, A. Poley, A. Policicchio, R. Polifka, A. Polini, C. S. Pollard, V. Polychronakos, K. Pommès, D. Ponomarenko, L. Pontecorvo, G. A. Popeneciu, A. Poppleton, S. Pospisil, K. Potamianos, I. N. Potrap, C. J. Potter, G. Poulard, T. Poulsen, J. Poveda, M. E. Pozo Astigarraga, P. Pralavorio, A. Pranko, S. Prell, D. Price, M. Primavera, S. Prince, N. Proklova, K. Prokofiev, F. Prokoshin, S. Protopopescu, J. Proudfoot, M. Przybycien, A. Puri, P. Puzo, J. Qian, G. Qin, Y. Qin, A. Quadt, M. Queitsch-Maitland, D. Quilty, S. Raddum, V. Radeka, V. Radescu, S. K. Radhakrishnan, P. Radloff, P. Rados, F. Ragusa, G. Rahal, J. A. Raine, S. Rajagopalan, C. Rangel-Smith, T. Rashid, S. Raspopov, M. G. Ratti, D. M. Rauch, F. Rauscher, S. Rave, I. Ravinovich, J. H. Rawling, M. Raymond, A. L. Read, N. P. Readioff, M. Reale, D. M. Rebuzzi, A. Redelbach, G. Redlinger, R. Reece, R. G. Reed, K. Reeves, L. Rehnisch, J. Reichert, A. Reiss, C. Rembser, H. Ren, M. Rescigno, S. Resconi, E. D. Resseguie, S. Rettie, E. Reynolds, O. L. Rezanova, P. Reznicek, R. Rezvani, R. Richter, S. Richter, E. Richter-Was, O. Ricken, M. Ridel, P. Rieck, C. J. Riegel, J. Rieger, O. Rifki, M. Rijssenbeek, A. Rimoldi, M. Rimoldi, L. Rinaldi, G. Ripellino, B. Ristić, E. Ritsch, I. Riu, F. Rizatdinova, E. Rizvi, C. Rizzi, R. T. Roberts, S. H. Robertson, A. Robichaud-Veronneau, D. Robinson, J. E. M. Robinson, A. Robson, E. Rocco, C. Roda, Y. Rodina, S. Rodriguez Bosca, A. Rodriguez Perez, D. Rodriguez Rodriguez, S. Roe, C. S. Rogan, O. Røhne, J. Roloff, A. Romaniouk, M. Romano, S. M. Romano Saez, E. Romero Adam, N. Rompotis, M. Ronzani, L. Roos, S. Rosati, K. Rosbach, P. Rose, N.-A. Rosien, E. Rossi, L. P. Rossi, J. H. N. Rosten, R. Rosten, M. Rotaru, J. Rothberg, D. Rousseau, A. Rozanov, Y. Rozen, X. Ruan, F. Rubbo, F. Rühr, A. Ruiz-Martinez, Z. Rurikova, N. A. Rusakovich, H. L. Russell, J. P. Rutherfoord, N. Ruthmann, Y. F. Ryabov, M. Rybar, G. Rybkin, S. Ryu, A. Ryzhov, G. F. Rzehorz, A. F. Saavedra, G. Sabato, S. Sacerdoti, H.F-W. Sadrozinski, R. Sadykov, F. Safai Tehrani, P. Saha, M. Sahinsoy, M. Saimpert, M. Saito, T. Saito, H. Sakamoto, Y. Sakurai, G. Salamanna, J. E. Salazar Loyola, D. Salek, P. H. Sales De Bruin, D. Salihagic, A. Salnikov, J. Salt, D. Salvatore, F. Salvatore, A. Salvucci, A. Salzburger, D. Sammel, D. Sampsonidis, D. Sampsonidou, J. Sánchez, V. Sanchez Martinez, A. Sanchez Pineda, H. Sandaker, R. L. Sandbach, C. O. Sander, M. Sandhoff, C. Sandoval, D. P. C. Sankey, M. Sannino, Y. Sano, A. Sansoni, C. Santoni, H. Santos, I. Santoyo Castillo, A. Sapronov, J. G. Saraiva, B. Sarrazin, O. Sasaki, K. Sato, E. Sauvan, G. Savage, P. Savard, N. Savic, C. Sawyer, L. Sawyer, J. Saxon, C. Sbarra, A. Sbrizzi, T. Scanlon, D. A. Scannicchio, M. Scarcella, J. Schaarschmidt, P. Schacht, B. M. Schachtner, D. Schaefer, L. Schaefer, R. Schaefer, J. Schaeffer, S. Schaepe, S. Schaetzel, U. Schäfer, A. C. Schaffer, D. Schaile, R. D. Schamberger, V. A. Schegelsky, D. Scheirich, M. Schernau, C. Schiavi, S. Schier, L. K. Schildgen, C. Schillo, M. Schioppa, S. Schlenker, K. R. Schmidt-Sommerfeld, K. Schmieden, C. Schmitt, S. Schmitt, S. Schmitz, U. Schnoor, L. Schoeffel, A. Schoening, B. D. Schoenrock, E. Schopf, M. Schott, J. F. P. Schouwenberg, J. Schovancova, S. Schramm, N. Schuh, A. Schulte, M. J. Schultens, H.-C. Schultz-Coulon, H. Schulz, M. Schumacher, B. A. Schumm, Ph. Schune, A. Schwartzman, T. A. Schwarz, H. Schweiger, Ph. Schwemling, R. Schwienhorst, J. Schwindling, A. Sciandra, G. Sciolla, M. Scornajenghi, F. Scuri, F. Scutti, J. Searcy, P. Seema, S. C. Seidel, A. Seiden, J. M. Seixas, G. Sekhniaidze, K. Sekhon, S. J. Sekula, N. Semprini-Cesari, S. Senkin, C. Serfon, L. Serin, L. Serkin, M. Sessa, R. Seuster, H. Severini, T. Sfiligoj, F. Sforza, A. Sfyrla, E. Shabalina, N. W. Shaikh, L. Y. Shan, R. Shang, J. T. Shank, M. Shapiro, P. B. Shatalov, K. Shaw, S. M. Shaw, A. Shcherbakova, C. Y. Shehu, Y. Shen, N. Sherafati, P. Sherwood, L. Shi, S. Shimizu, C. O. Shimmin, M. Shimojima, I. P. J. Shipsey, S. Shirabe, M. Shiyakova, J. Shlomi, A. Shmeleva, D. Shoaleh Saadi, M. J. Shochet, S. Shojaii, D. R. Shope, S. Shrestha, E. Shulga, M. A. Shupe, P. Sicho, A. M. Sickles, P. E. Sidebo, E. Sideras Haddad, O. Sidiropoulou, A. Sidoti, F. Siegert, Dj. Sijacki, J. Silva, S. B. Silverstein, V. Simak, L. Simic, S. Simion, E. Simioni, B. Simmons, M. Simon, P. Sinervo, N. B. Sinev, M. Sioli, G. Siragusa, I. Siral, S. Yu. Sivoklokov, J. Sjölin, M. B. Skinner, P. Skubic, M. Slater, T. Slavicek, M. Slawinska, K. Sliwa, R. Slovak, V. Smakhtin, B. H. Smart, J. Smiesko, N. Smirnov, S. Yu. Smirnov, Y. Smirnov, L. N. Smirnova, O. Smirnova, J. W. Smith, M. N. K. Smith, R. W. Smith, M. Smizanska, K. Smolek, A. A. Snesarev, I. M. Snyder, S. Snyder, R. Sobie, F. Socher, A. Soffer, A. Søgaard, D. A. Soh, G. Sokhrannyi, C. A. Solans Sanchez, M. Solar, E. Yu. Soldatov, U. Soldevila, A. A. Solodkov, A. Soloshenko, O. V. Solovyanov, V. Solovyev, P. Sommer, H. Son, A. Sopczak, D. Sosa, C. L. Sotiropoulou, R. Soualah, A. M. Soukharev, D. South, B. C. Sowden, S. Spagnolo, M. Spalla, M. Spangenberg, F. Spanò, D. Sperlich, F. Spettel, T. M. Spieker, R. Spighi, G. Spigo, L. A. Spiller, M. Spousta, R. D. St. Denis, A. Stabile, R. Stamen, S. Stamm, E. Stanecka, R. W. Stanek, C. Stanescu, M. M. Stanitzki, B. S. Stapf, S. Stapnes, E. A. Starchenko, G. H. Stark, J. Stark, S. H Stark, P. Staroba, P. Starovoitov, S. Stärz, R. Staszewski, P. Steinberg, B. Stelzer, H. J. Stelzer, O. Stelzer-Chilton, H. Stenzel, G. A. Stewart, M. C. Stockton, M. Stoebe, G. Stoicea, P. Stolte, S. Stonjek, A. R. Stradling, A. Straessner, M. E. Stramaglia, J. Strandberg, S. Strandberg, M. Strauss, P. Strizenec, R. Ströhmer, D. M. Strom, R. Stroynowski, A. Strubig, S. A. Stucci, B. Stugu, N. A. Styles, D. Su, J. Su, S. Suchek, Y. Sugaya, M. Suk, V. V. Sulin, DMS Sultan, S. Sultansoy, T. Sumida, S. Sun, X. Sun, K. Suruliz, C. J. E. Suster, M. R. Sutton, S. Suzuki, M. Svatos, M. Swiatlowski, S. P. Swift, I. Sykora, T. Sykora, D. Ta, K. Tackmann, J. Taenzer, A. Taffard, R. Tafirout, E. Tahirovic, N. Taiblum, H. Takai, R. Takashima, E. H. Takasugi, T. Takeshita, Y. Takubo, M. Talby, A. A. Talyshev, J. Tanaka, M. Tanaka, R. Tanaka, S. Tanaka, R. Tanioka, B. B. Tannenwald, S. Tapia Araya, S. Tapprogge, S. Tarem, G. F. Tartarelli, P. Tas, M. Tasevsky, T. Tashiro, E. Tassi, A. Tavares Delgado, Y. Tayalati, A. C. Taylor, G. N. Taylor, P. T. E. Taylor, W. Taylor, P. Teixeira-Dias, D. Temple, H. Ten Kate, P. K. Teng, J. J. Teoh, F. Tepel, S. Terada, K. Terashi, J. Terron, S. Terzo, M. Testa, R. J. Teuscher, T. Theveneaux-Pelzer, F. Thiele, J. P. Thomas, J. Thomas-Wilsker, P. D. Thompson, A. S. Thompson, L. A. Thomsen, E. Thomson, M. J. Tibbetts, R. E. Ticse Torres, V. O. Tikhomirov, Yu. A. Tikhonov, S. Timoshenko, P. Tipton, S. Tisserant, K. Todome, S. Todorova-Nova, S. Todt, J. Tojo, S. Tokár, K. Tokushuku, E. Tolley, L. Tomlinson, M. Tomoto, L. Tompkins, K. Toms, B. Tong, P. Tornambe, E. Torrence, H. Torres, E. Torró Pastor, J. Toth, F. Touchard, D. R. Tovey, C. J. Treado, T. Trefzger, F. Tresoldi, A. Tricoli, I. M. Trigger, S. Trincaz-Duvoid, M. F. Tripiana, W. Trischuk, B. Trocmé, A. Trofymov, C. Troncon, M. Trottier-McDonald, M. Trovatelli, L. Truong, M. Trzebinski, A. Trzupek, K. W. Tsang, J.C-L. Tseng, P. V. Tsiareshka, G. Tsipolitis, N. Tsirintanis, S. Tsiskaridze, V. Tsiskaridze, E. G. Tskhadadze, K. M. Tsui, I. I. Tsukerman, V. Tsulaia, S. Tsuno, D. Tsybychev, Y. Tu, A. Tudorache, V. Tudorache, T. T. Tulbure, A. N. Tuna, S. A. Tupputi, S. Turchikhin, D. Turgeman, I. Turk Cakir, R. Turra, P. M. Tuts, G. Ucchielli, I. Ueda, M. Ughetto, F. Ukegawa, G. Unal, A. Undrus, G. Unel, F. C. Ungaro, Y. Unno, C. Unverdorben, J. Urban, P. Urquijo, P. Urrejola, G. Usai, J. Usui, L. Vacavant, V. Vacek, B. Vachon, K. O. H. Vadla, A. Vaidya, C. Valderanis, E. Valdes Santurio, S. Valentinetti, A. Valero, L. Valéry, S. Valkar, A. Vallier, J. A. Valls Ferrer, W. Van Den Wollenberg, H. van der Graaf, P. van Gemmeren, J. Van Nieuwkoop, I. van Vulpen, M. C. van Woerden, M. Vanadia, W. Vandelli, A. Vaniachine, P. Vankov, G. Vardanyan, R. Vari, E. W. Varnes, C. Varni, T. Varol, D. Varouchas, A. Vartapetian, K. E. Varvell, J. G. Vasquez, G. A. Vasquez, F. Vazeille, T. Vazquez Schroeder, J. Veatch, V. Veeraraghavan, L. M. Veloce, F. Veloso, S. Veneziano, A. Ventura, M. Venturi, N. Venturi, A. Venturini, V. Vercesi, M. Verducci, W. Verkerke, A. T. Vermeulen, J. C. Vermeulen, M. C. Vetterli, N. Viaux Maira, O. Viazlo, I. Vichou, T. Vickey, O. E. Vickey Boeriu, G. H. A. Viehhauser, S. Viel, L. Vigani, M. Villa, M. Villaplana Perez, E. Vilucchi, M. G. Vincter, V. B. Vinogradov, A. Vishwakarma, C. Vittori, I. Vivarelli, S. Vlachos, M. Vogel, P. Vokac, G. Volpi, H. von der Schmitt, E. von Toerne, V. Vorobel, K. Vorobev, M. Vos, R. Voss, J. H. Vossebeld, N. Vranjes, M. Vranjes Milosavljevic, V. Vrba, M. Vreeswijk, R. Vuillermet, I. Vukotic, P. Wagner, W. Wagner, J. Wagner-Kuhr, H. Wahlberg, S. Wahrmund, J. Wakabayashi, J. Walder, R. Walker, W. Walkowiak, V. Wallangen, C. Wang, C. Wang, F. Wang, H. Wang, H. Wang, J. Wang, J. Wang, Q. Wang, R. Wang, S. M. Wang, T. Wang, W. Wang, W. Wang, Z. Wang, C. Wanotayaroj, A. Warburton, C. P. Ward, D. R. Wardrope, A. Washbrook, P. M. Watkins, A. T. Watson, M. F. Watson, G. Watts, S. Watts, B. M. Waugh, A. F. Webb, S. Webb, M. S. Weber, S. W. Weber, S. A. Weber, J. S. Webster, A. R. Weidberg, B. Weinert, J. Weingarten, M. Weirich, C. Weiser, H. Weits, P. S. Wells, T. Wenaus, T. Wengler, S. Wenig, N. Wermes, M. D. Werner, P. Werner, M. Wessels, K. Whalen, N. L. Whallon, A. M. Wharton, A. S. White, A. White, M. J. White, R. White, D. Whiteson, B. W. Whitmore, F. J. Wickens, W. Wiedenmann, M. Wielers, C. Wiglesworth, L. A. M. Wiik-Fuchs, A. Wildauer, F. Wilk, H. G. Wilkens, H. H. Williams, S. Williams, C. Willis, S. Willocq, J. A. Wilson, I. Wingerter-Seez, E. Winkels, F. Winklmeier, O. J. Winston, B. T. Winter, M. Wittgen, M. Wobisch, T. M. H. Wolf, R. Wolff, M. W. Wolter, H. Wolters, V. W. S. Wong, S. D. Worm, B. K. Wosiek, J. Wotschack, K. W. Wozniak, M. Wu, S. L. Wu, X. Wu, Y. Wu, T. R. Wyatt, B. M. Wynne, S. Xella, Z. Xi, L. Xia, D. Xu, L. Xu, T. Xu, B. Yabsley, S. Yacoob, D. Yamaguchi, Y. Yamaguchi, A. Yamamoto, S. Yamamoto, T. Yamanaka, M. Yamatani, K. Yamauchi, Y. Yamazaki, Z. Yan, H. Yang, H. Yang, Y. Yang, Z. Yang, W-M. Yao, Y. C. Yap, Y. Yasu, E. Yatsenko, K. H. Yau Wong, J. Ye, S. Ye, I. Yeletskikh, E. Yigitbasi, E. Yildirim, K. Yorita, K. Yoshihara, C. Young, C. J. S. Young, J. Yu, J. Yu, S. P. Y. Yuen, I. Yusuff, B. Zabinski, G. Zacharis, R. Zaidan, A. M. Zaitsev, N. Zakharchuk, J. Zalieckas, A. Zaman, S. Zambito, D. Zanzi, C. Zeitnitz, G. Zemaityte, A. Zemla, J. C. Zeng, Q. Zeng, O. Zenin, T. Ženiš, D. Zerwas, D. Zhang, F. Zhang, G. Zhang, H. Zhang, J. Zhang, L. Zhang, L. Zhang, M. Zhang, P. Zhang, R. Zhang, R. Zhang, X. Zhang, Y. Zhang, Z. Zhang, X. Zhao, Y. Zhao, Z. Zhao, A. Zhemchugov, B. Zhou, C. Zhou, L. Zhou, M. Zhou, M. Zhou, N. Zhou, C. G. Zhu, H. Zhu, J. Zhu, Y. Zhu, X. Zhuang, K. Zhukov, A. Zibell, D. Zieminska, N. I. Zimine, C. Zimmermann, S. Zimmermann, Z. Zinonos, M. Zinser, M. Ziolkowski, L. Živković, G. Zobernig, A. Zoccoli, R. Zou, M. zur Nedden, L. Zwalinski, ATLAS Collaboration

**Affiliations:** 1 Department of Physics, University of Adelaide, Adelaide, Australia; 2 Physics Department, SUNY Albany, Albany, NY USA; 3 Department of Physics, University of Alberta, Edmonton, AB Canada; 4 Department of Physics, Ankara University, Ankara, Turkey; 5 Istanbul Aydin University, Istanbul, Turkey; 6 Division of Physics, TOBB University of Economics and Technology, Ankara, Turkey; 7 LAPP, CNRS/IN2P3 and Université Savoie Mont Blanc, Annecy-le-Vieux, France; 8 High Energy Physics Division, Argonne National Laboratory, Argonne, IL USA; 9 Department of Physics, University of Arizona, Tucson, AZ USA; 10 Department of Physics, The University of Texas at Arlington, Arlington, TX USA; 11 Physics Department, National and Kapodistrian University of Athens, Athens, Greece; 12 Physics Department, National Technical University of Athens, Zografou, Greece; 13 Department of Physics, The University of Texas at Austin, Austin, TX USA; 14 Institute of Physics, Azerbaijan Academy of Sciences, Baku, Azerbaijan; 15 Institut de Física d’Altes Energies (IFAE), The Barcelona Institute of Science and Technology, Barcelona, Spain; 16 Institute of Physics, University of Belgrade, Belgrade, Serbia; 17 Department for Physics and Technology, University of Bergen, Bergen, Norway; 18 Physics Division, Lawrence Berkeley National Laboratory and University of California, Berkeley, CA USA; 19 Department of Physics, Humboldt University, Berlin, Germany; 20 Albert Einstein Center for Fundamental Physics and Laboratory for High Energy Physics, University of Bern, Bern, Switzerland; 21 School of Physics and Astronomy, University of Birmingham, Birmingham, UK; 22 Department of Physics, Bogazici University, Istanbul, Turkey; 23 Department of Physics Engineering, Gaziantep University, Gaziantep, Turkey; 24 Faculty of Engineering and Natural Sciences, Istanbul Bilgi University, Istanbul, Turkey; 25 Faculty of Engineering and Natural Sciences, Bahcesehir University, Istanbul, Turkey; 26 Centro de Investigaciones, Universidad Antonio Narino, Bogotá, Colombia; 27 INFN Sezione di Bologna, Bologna, Italy; 28 Dipartimento di Fisica e Astronomia, Università di Bologna, Bologna, Italy; 29 Physikalisches Institut, University of Bonn, Bonn, Germany; 30 Department of Physics, Boston University, Boston, MA USA; 31 Department of Physics, Brandeis University, Waltham, MA USA; 32 Universidade Federal do Rio De Janeiro COPPE/EE/IF, Rio de Janeiro, Brazil; 33 Electrical Circuits Department, Federal University of Juiz de Fora (UFJF), Juiz de Fora, Brazil; 34 Federal University of Sao Joao del Rei (UFSJ), Sao Joao del Rei, Brazil; 35 Instituto de Fisica, Universidade de Sao Paulo, São Paulo, Brazil; 36 Physics Department, Brookhaven National Laboratory, Upton, NY USA; 37 Transilvania University of Brasov, Brasov, Romania; 38 Horia Hulubei National Institute of Physics and Nuclear Engineering, Bucharest, Romania; 39 Department of Physics, Alexandru Ioan Cuza University of Iasi, Iasi, Romania; 40 Physics Department, National Institute for Research and Development of Isotopic and Molecular Technologies, Cluj-Napoca, Romania; 41 University Politehnica Bucharest, Bucharest, Romania; 42 West University in Timisoara, Timisoara, Romania; 43 Departamento de Física, Universidad de Buenos Aires, Buenos Aires, Argentina; 44 Cavendish Laboratory, University of Cambridge, Cambridge, UK; 45 Department of Physics, Carleton University, Ottawa, ON Canada; 46 CERN, Geneva, Switzerland; 47 Enrico Fermi Institute, University of Chicago, Chicago, IL USA; 48 Departamento de Física, Pontificia Universidad Católica de Chile, Santiago, Chile; 49 Departamento de Física, Universidad Técnica Federico Santa María, Valparaiso, Chile; 50 Institute of High Energy Physics, Chinese Academy of Sciences, Beijing, China; 51 Department of Physics, Nanjing University, Nanjing, Jiangsu China; 52 Physics Department, Tsinghua University, Beijing, 100084 China; 53 University of Chinese Academy of Science (UCAS), Beijing, China; 54 Department of Modern Physics and State Key Laboratory of Particle Detection and Electronics, University of Science and Technology of China, Anhui, China; 55 School of Physics, Shandong University, Jinan, Shandong China; 56 Department of Physics and Astronomy, Key Laboratory for Particle Physics, Astrophysics and Cosmology, Ministry of Education, Shanghai Key Laboratory for Particle Physics and Cosmology, Shanghai Jiao Tong University, Shanghai (also at PKU-CHEP), Shanghai, China; 57 Université Clermont Auvergne, CNRS/IN2P3, LPC, Clermont-Ferrand, France; 58 Nevis Laboratory, Columbia University, Irvington, NY USA; 59 Niels Bohr Institute, University of Copenhagen, Copenhagen, Denmark; 60 INFN Gruppo Collegato di Cosenza, Laboratori Nazionali di Frascati, Frascati, Italy; 61 Dipartimento di Fisica, Università della Calabria, Rende, Italy; 62 Faculty of Physics and Applied Computer Science, AGH University of Science and Technology, Kraków, Poland; 63 Marian Smoluchowski Institute of Physics, Jagiellonian University, Kraków, Poland; 64 Institute of Nuclear Physics, Polish Academy of Sciences, Kraków, Poland; 65 Physics Department, Southern Methodist University, Dallas, TX USA; 66 Physics Department, University of Texas at Dallas, Richardson, TX USA; 67 DESY, Hamburg and Zeuthen, Germany; 68 Lehrstuhl für Experimentelle Physik IV, Technische Universität Dortmund, Dortmund, Germany; 69 Institut für Kern- und Teilchenphysik, Technische Universität Dresden, Dresden, Germany; 70 Department of Physics, Duke University, Durham, NC USA; 71 SUPA-School of Physics and Astronomy, University of Edinburgh, Edinburgh, UK; 72 INFN e Laboratori Nazionali di Frascati, Frascati, Italy; 73 Fakultät für Mathematik und Physik, Albert-Ludwigs-Universität, Freiburg, Germany; 74 Departement de Physique Nucleaire et Corpusculaire, Université de Genève, Geneva, Switzerland; 75 INFN Sezione di Genova, Genoa, Italy; 76 Dipartimento di Fisica, Università di Genova, Genoa, Italy; 77 E. Andronikashvili Institute of Physics, Iv. Javakhishvili Tbilisi State University, Tbilisi, Georgia; 78 High Energy Physics Institute, Tbilisi State University, Tbilisi, Georgia; 79 II Physikalisches Institut, Justus-Liebig-Universität Giessen, Giessen, Germany; 80 SUPA-School of Physics and Astronomy, University of Glasgow, Glasgow, UK; 81 II Physikalisches Institut, Georg-August-Universität, Göttingen, Germany; 82 Laboratoire de Physique Subatomique et de Cosmologie, Université Grenoble-Alpes, CNRS/IN2P3, Grenoble, France; 83 Laboratory for Particle Physics and Cosmology, Harvard University, Cambridge, MA USA; 84 Kirchhoff-Institut für Physik, Ruprecht-Karls-Universität Heidelberg, Heidelberg, Germany; 85 Physikalisches Institut, Ruprecht-Karls-Universität Heidelberg, Heidelberg, Germany; 86 Faculty of Applied Information Science, Hiroshima Institute of Technology, Hiroshima, Japan; 87 Department of Physics, The Chinese University of Hong Kong, Shatin, NT Hong Kong; 88 Department of Physics, The University of Hong Kong, Hong Kong, China; 89 Department of Physics and Institute for Advanced Study, The Hong Kong University of Science and Technology, Clear Water Bay, Kowloon, Hong Kong, China; 90 Department of Physics, National Tsing Hua University, Taiwan, Taiwan; 91 Department of Physics, Indiana University, Bloomington, IN USA; 92 Institut für Astro- und Teilchenphysik, Leopold-Franzens-Universität, Innsbruck, Austria; 93 University of Iowa, Iowa City, IA USA; 94 Department of Physics and Astronomy, Iowa State University, Ames, IA USA; 95 Joint Institute for Nuclear Research, JINR Dubna, Dubna, Russia; 96 KEK, High Energy Accelerator Research Organization, Tsukuba, Japan; 97 Graduate School of Science, Kobe University, Kobe, Japan; 98 Faculty of Science, Kyoto University, Kyoto, Japan; 99 Kyoto University of Education, Kyoto, Japan; 100 Research Center for Advanced Particle Physics and Department of Physics, Kyushu University, Fukuoka, Japan; 101 Instituto de Física La Plata, Universidad Nacional de La Plata and CONICET, La Plata, Argentina; 102 Physics Department, Lancaster University, Lancaster, UK; 103 INFN Sezione di Lecce, Lecce, Italy; 104 Dipartimento di Matematica e Fisica, Università del Salento, Lecce, Italy; 105 Oliver Lodge Laboratory, University of Liverpool, Liverpool, UK; 106 Department of Experimental Particle Physics, Jožef Stefan Institute and Department of Physics, University of Ljubljana, Ljubljana, Slovenia; 107 School of Physics and Astronomy, Queen Mary University of London, London, UK; 108 Department of Physics, Royal Holloway University of London, Surrey, UK; 109 Department of Physics and Astronomy, University College London, London, UK; 110 Louisiana Tech University, Ruston, LA USA; 111 Laboratoire de Physique Nucléaire et de Hautes Energies, UPMC and Université Paris-Diderot and CNRS/IN2P3, Paris, France; 112 Fysiska institutionen, Lunds universitet, Lund, Sweden; 113 Departamento de Fisica Teorica C-15, Universidad Autonoma de Madrid, Madrid, Spain; 114 Institut für Physik, Universität Mainz, Mainz, Germany; 115 School of Physics and Astronomy, University of Manchester, Manchester, UK; 116 CPPM, Aix-Marseille Université and CNRS/IN2P3, Marseille, France; 117 Department of Physics, University of Massachusetts, Amherst, MA USA; 118 Department of Physics, McGill University, Montreal, QC Canada; 119 School of Physics, University of Melbourne, Victoria, Australia; 120 Department of Physics, The University of Michigan, Ann Arbor, MI USA; 121 Department of Physics and Astronomy, Michigan State University, East Lansing, MI USA; 122 INFN Sezione di Milano, Milan, Italy; 123 Dipartimento di Fisica, Università di Milano, Milan, Italy; 124 B.I. Stepanov Institute of Physics, National Academy of Sciences of Belarus, Minsk, Republic of Belarus; 125 Research Institute for Nuclear Problems of Byelorussian State University, Minsk, Republic of Belarus; 126 Group of Particle Physics, University of Montreal, Montreal, QC Canada; 127 P.N. Lebedev Physical Institute of the Russian Academy of Sciences, Moscow, Russia; 128 Institute for Theoretical and Experimental Physics (ITEP), Moscow, Russia; 129 National Research Nuclear University MEPhI, Moscow, Russia; 130 D.V. Skobeltsyn Institute of Nuclear Physics, M.V. Lomonosov Moscow State University, Moscow, Russia; 131 Fakultät für Physik, Ludwig-Maximilians-Universität München, Munich, Germany; 132 Max-Planck-Institut für Physik (Werner-Heisenberg-Institut), Munich, Germany; 133 Nagasaki Institute of Applied Science, Nagasaki, Japan; 134 Graduate School of Science and Kobayashi-Maskawa Institute, Nagoya University, Nagoya, Japan; 135 INFN Sezione di Napoli, Naples, Italy; 136 Dipartimento di Fisica, Università di Napoli, Naples, Italy; 137 Department of Physics and Astronomy, University of New Mexico, Albuquerque, NM USA; 138 Institute for Mathematics, Astrophysics and Particle Physics, Radboud University Nijmegen/Nikhef, Nijmegen, The Netherlands; 139 Nikhef National Institute for Subatomic Physics and University of Amsterdam, Amsterdam, The Netherlands; 140 Department of Physics, Northern Illinois University, DeKalb, IL USA; 141 Budker Institute of Nuclear Physics, SB RAS, Novosibirsk, Russia; 142 Department of Physics, New York University, New York, NY USA; 143 Ohio State University, Columbus, OH USA; 144 Faculty of Science, Okayama University, Okayama, Japan; 145 Homer L. Dodge Department of Physics and Astronomy, University of Oklahoma, Norman, OK USA; 146 Department of Physics, Oklahoma State University, Stillwater, OK USA; 147 Palacký University, RCPTM, Olomouc, Czech Republic; 148 Center for High Energy Physics, University of Oregon, Eugene, OR USA; 149 LAL, Univ. Paris-Sud, CNRS/IN2P3, Université Paris-Saclay, Orsay, France; 150 Graduate School of Science, Osaka University, Osaka, Japan; 151 Department of Physics, University of Oslo, Oslo, Norway; 152 Department of Physics, Oxford University, Oxford, UK; 153 INFN Sezione di Pavia, Pavia, Italy; 154 Dipartimento di Fisica, Università di Pavia, Pavia, Italy; 155 Department of Physics, University of Pennsylvania, Philadelphia, PA USA; 156 National Research Centre “Kurchatov Institute” B.P. Konstantinov Petersburg Nuclear Physics Institute, St. Petersburg, Russia; 157 INFN Sezione di Pisa, Pisa, Italy; 158 Dipartimento di Fisica E. Fermi, Università di Pisa, Pisa, Italy; 159 Department of Physics and Astronomy, University of Pittsburgh, Pittsburgh, PA USA; 160 Laboratório de Instrumentação e Física Experimental de Partículas-LIP, Lisbon, Portugal; 161 Faculdade de Ciências, Universidade de Lisboa, Lisbon, Portugal; 162 Department of Physics, University of Coimbra, Coimbra, Portugal; 163 Centro de Física Nuclear da Universidade de Lisboa, Lisbon, Portugal; 164 Departamento de Fisica, Universidade do Minho, Braga, Portugal; 165 Departamento de Fisica Teorica y del Cosmos, Universidad de Granada, Granada, Spain; 166 Dep Fisica and CEFITEC of Faculdade de Ciencias e Tecnologia, Universidade Nova de Lisboa, Caparica, Portugal; 167 Institute of Physics, Academy of Sciences of the Czech Republic, Prague, Czech Republic; 168 Czech Technical University in Prague, Prague, Czech Republic; 169 Faculty of Mathematics and Physics, Charles University, Prague, Czech Republic; 170 State Research Center Institute for High Energy Physics (Protvino), NRC KI, Protvino, Russia; 171 Particle Physics Department, Rutherford Appleton Laboratory, Didcot, UK; 172 INFN Sezione di Roma, Rome, Italy; 173 Dipartimento di Fisica, Sapienza Università di Roma, Rome, Italy; 174 INFN Sezione di Roma Tor Vergata, Rome, Italy; 175 Dipartimento di Fisica, Università di Roma Tor Vergata, Rome, Italy; 176 INFN Sezione di Roma Tre, Rome, Italy; 177 Dipartimento di Matematica e Fisica, Università Roma Tre, Rome, Italy; 178 Faculté des Sciences Ain Chock, Réseau Universitaire de Physique des Hautes Energies-Université Hassan II, Casablanca, Morocco; 179 Centre National de l’Energie des Sciences Techniques Nucleaires, Rabat, Morocco; 180 Faculté des Sciences Semlalia, Université Cadi Ayyad, LPHEA-Marrakech, Marrakech, Morocco; 181 Faculté des Sciences, Université Mohamed Premier and LPTPM, Oujda, Morocco; 182 Faculté des Sciences, Université Mohammed V, Rabat, Morocco; 183 DSM/IRFU (Institut de Recherches sur les Lois Fondamentales de l’Univers), CEA Saclay (Commissariat à l’Energie Atomique et aux Energies Alternatives), Gif-sur-Yvette, France; 184 Santa Cruz Institute for Particle Physics, University of California Santa Cruz, Santa Cruz, CA USA; 185 Department of Physics, University of Washington, Seattle, WA USA; 186 Department of Physics and Astronomy, University of Sheffield, Sheffield, UK; 187 Department of Physics, Shinshu University, Nagano, Japan; 188 Department Physik, Universität Siegen, Siegen, Germany; 189 Department of Physics, Simon Fraser University, Burnaby, BC Canada; 190 SLAC National Accelerator Laboratory, Stanford, CA USA; 191 Faculty of Mathematics, Physics and Informatics, Comenius University, Bratislava, Slovak Republic; 192 Department of Subnuclear Physics, Institute of Experimental Physics of the Slovak Academy of Sciences, Kosice, Slovak Republic; 193 Department of Physics, University of Cape Town, Cape Town, South Africa; 194 Department of Physics, University of Johannesburg, Johannesburg, South Africa; 195 School of Physics, University of the Witwatersrand, Johannesburg, South Africa; 196 Department of Physics, Stockholm University, Stockholm, Sweden; 197 The Oskar Klein Centre, Stockholm, Sweden; 198 Physics Department, Royal Institute of Technology, Stockholm, Sweden; 199 Departments of Physics and Astronomy and Chemistry, Stony Brook University, Stony Brook, NY USA; 200 Department of Physics and Astronomy, University of Sussex, Brighton, UK; 201 School of Physics, University of Sydney, Sydney, Australia; 202 Institute of Physics, Academia Sinica, Taipei, Taiwan; 203 Department of Physics, Technion: Israel Institute of Technology, Haifa, Israel; 204 Raymond and Beverly Sackler School of Physics and Astronomy, Tel Aviv University, Tel Aviv, Israel; 205 Department of Physics, Aristotle University of Thessaloniki, Thessaloníki, Greece; 206 International Center for Elementary Particle Physics and Department of Physics, The University of Tokyo, Tokyo, Japan; 207 Graduate School of Science and Technology, Tokyo Metropolitan University, Tokyo, Japan; 208 Department of Physics, Tokyo Institute of Technology, Tokyo, Japan; 209 Tomsk State University, Tomsk, Russia; 210 Department of Physics, University of Toronto, Toronto, ON Canada; 211 INFN-TIFPA, Trento, Italy; 212 University of Trento, Trento, Italy; 213 TRIUMF, Vancouver, BC Canada; 214 Department of Physics and Astronomy, York University, Toronto, ON Canada; 215 Faculty of Pure and Applied Sciences, and Center for Integrated Research in Fundamental Science and Engineering, University of Tsukuba, Tsukuba, Japan; 216 Department of Physics and Astronomy, Tufts University, Medford, MA USA; 217 Department of Physics and Astronomy, University of California Irvine, Irvine, CA USA; 218 INFN Gruppo Collegato di Udine, Sezione di Trieste, Udine, Italy; 219 ICTP, Trieste, Italy; 220 Dipartimento di Chimica, Fisica e Ambiente, Università di Udine, Udine, Italy; 221 Department of Physics and Astronomy, University of Uppsala, Uppsala, Sweden; 222 Department of Physics, University of Illinois, Urbana, IL USA; 223 Instituto de Fisica Corpuscular (IFIC), Centro Mixto Universidad de Valencia-CSIC, Valencia, Spain; 224 Department of Physics, University of British Columbia, Vancouver, BC Canada; 225 Department of Physics and Astronomy, University of Victoria, Victoria, BC Canada; 226 Department of Physics, University of Warwick, Coventry, UK; 227 Waseda University, Tokyo, Japan; 228 Department of Particle Physics, The Weizmann Institute of Science, Rehovot, Israel; 229 Department of Physics, University of Wisconsin, Madison, WI USA; 230 Fakultät für Physik und Astronomie, Julius-Maximilians-Universität, Würzburg, Germany; 231 Fakultät für Mathematik und Naturwissenschaften, Fachgruppe Physik, Bergische Universität Wuppertal, Wuppertal, Germany; 232 Department of Physics, Yale University, New Haven, CT USA; 233 Yerevan Physics Institute, Yerevan, Armenia; 234 Centre de Calcul de l’Institut National de Physique Nucléaire et de Physique des Particules (IN2P3), Villeurbanne, France; 235 Academia Sinica Grid Computing, Institute of Physics, Academia Sinica, Taipei, Taiwan; 236 CERN, 1211 Geneva 23, Switzerland

## Abstract

A search for massive coloured resonances which are pair-produced and decay into two jets is presented. The analysis uses 36.7 fb$^{-1}$ of $\sqrt{s}$ = 13 TeV *pp* collision data recorded by the ATLAS experiment at the LHC in 2015 and 2016. No significant deviation from the background prediction is observed. Results are interpreted in a SUSY simplified model where the lightest supersymmetric particle is the top squark, $\tilde{t}$, which decays promptly into two quarks through *R*-parity-violating couplings. Top squarks with masses in the range $100~\text {GeV}<m_{\tilde{t}}<410$ $\text {GeV}$ are excluded at 95% confidence level. If the decay is into a *b*-quark and a light quark, a dedicated selection requiring two *b*-tags is used to exclude masses in the ranges $100~\text {GeV}<m_{\tilde{t}}<470$ $\text {GeV}$ and $480~\text {GeV}<m_{\tilde{t}}<610$ $\text {GeV}$. Additional limits are set on the pair-production of massive colour-octet resonances.

## Introduction

Massive coloured particles decaying into quarks and gluons are predicted in several extensions of the Standard Model (SM). At hadron colliders, the search for new phenomena in fully hadronic final states, without missing transverse momentum, is experimentally challenging due to the very large SM multijet production cross-section. This paper describes a search for pair-produced particles each decaying into two jets using 36.7 fb$^{-1}$ of $\sqrt{s}=13$ $\text {TeV}$ proton–proton (*pp*) collision data recorded in 2015 and 2016 by the ATLAS experiment at the Large Hadron Collider (LHC).

Supersymmetry (SUSY) [1–7] is a generalisation of the Poincaré symmetry group that relates fermionic and bosonic degrees of freedom. In the generic superpotential, Yukawa couplings can lead to baryon- and lepton-number violation:

$$\begin{aligned} \mathcal {W}_{\text {RPV}} = \lambda _{ijk}L_iL_j\overline{E}_k+\lambda '_{ijk}L_i Q_j\overline{D}_k+\lambda ''_{ijk}\overline{U}_i\overline{D}_j\overline{D}_k + \kappa _iL_iH_u, \end{aligned}$$

where *i*, *j*, and *k* are quark and lepton generation indices. The $L_i$ and $Q_i$ represent the lepton and quark $\mathrm {SU}(2)_\mathrm {L}$ doublet superfields and $H_u$ the Higgs superfield that couples to up-type quarks. The $\bar{E}_i$, $\bar{D}_i$, and $\bar{U}_i$ are the lepton, down-type quark and up-type quark $\mathrm {SU}(2)_\mathrm {L}$ singlet superfields, respectively. For each term the couplings are $\lambda$, $\lambda '$, $\lambda ''$, as well as $\kappa$ which is a dimensional mass parameter. The $\lambda$ and $\lambda ''$ couplings are antisymmetric in the exchange of $i\rightarrow j$ and $j\rightarrow k$, respectively. While these terms in many scenarios are removed by imposing an additional $Z_2$ symmetry (*R*-parity) [8], the possibility that at least some of these *R*-parity-violating (RPV) couplings are not zero is not ruled out experimentally [9, 10]. This family of models leads to unique collider signatures which can escape conventional searches for *R*-parity-conserving SUSY.

Naturalness arguments [11, 12] suggest that higgsinos and top squarks^1^ (stops) should be light, with masses below a $\text {TeV}$ [13, 14]. Third-generation squarks in *R*-parity-conserving scenarios, and top squarks in particular, have been the subject of a thorough programme of searches at the LHC [15–22].

If the top squark decays through RPV couplings, however, the existing bounds on its mass can be significantly relaxed [23–26]. Indirect experimental constraints [27] on the sizes of each of the $\lambda ''$ couplings are primarily valid for low squark mass and for first- and second-generation couplings.

**Fig. 1:**
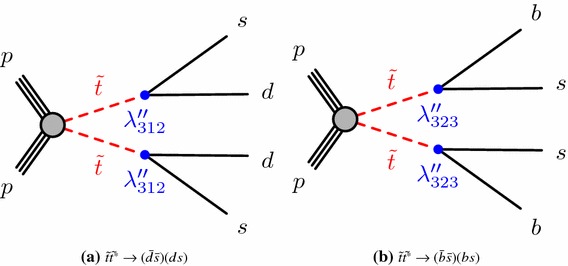
Diagrams depicting the direct pair-production of top squarks through strong interactions, with decays into a *d*- and an *s*-quark (left) or into a *b*- and an *s*-quark (right) through the $\lambda ''$ *R*-parity-violating couplings, indicated by the blue dots

This search targets a model where the top squark is the lightest supersymmetric particle and decays through baryon-number-violating RPV $\lambda ''$ couplings, $\tilde{t}\rightarrow \bar{q}_j\bar{q}_k$. The couplings are assumed to be sufficiently large for the decays to be prompt, but small enough to neglect the single-top-squark resonant production through RPV couplings. Top squarks are then produced through strong interactions with cross-sections that do not depend on the specific assumptions in the SUSY model. For two specific choices of couplings, the process considered is schematically depicted in Fig. 1.

In models with extended SUSY, colour-octet states can arise as scalar partners of a Dirac gluino [28–31]. These scalar gluons (or sgluons) are mostly produced in pairs, and decay into two quarks or two gluons.

Massive colour octet-resonances, generically referred to as colorons ($\rho$) [32, 33] are predicted in a wide range of other theories, including axigluon [34, 35] and topcolor [36], in vector-like confinement models [37, 38] and as Kaluza–Klein excitations of the gluons [39, 40]. Colorons can be pair-produced and decay into two jets, a scenario which leads to a four-jet final state.

Constraints on top squarks decaying through $\lambda ''$ couplings were first set by the ALEPH experiment at LEP [41], excluding at 95% confidence level (CL) masses below 80 $\text {GeV}$. The CDF experiment at the Tevatron [42], increased these limits to 100 $\text {GeV}$. Searches for pair-produced resonances in hadronic final states were performed at the LHC at 7 $\text {TeV}$ and 8 $\text {TeV}$ of centre-of-mass energy by both the ATLAS [43, 44] and CMS experiments [45, 46]. For decays including heavy-flavour jets in the final state, exclusion limits at 95% CL on the mass of the top squark in the ranges $100\, \text {GeV}\le m_{\tilde{t}}\le 320$ $\text {GeV}$ and $200\, \text {GeV}\le m_{\tilde{t}}\le 385$ $\text {GeV}$ have been reported by ATLAS [44] and CMS [46], respectively.

## ATLAS detector

The ATLAS detector [47] is a multi-purpose particle physics detector with a forward-backward symmetric cylindrical geometry and nearly $4\pi$ coverage in solid angle.^2^ The inner tracking detector consists of pixel and silicon microstrip detectors covering the pseudorapidity region $|\eta | < 2.5$, surrounded by a transition radiation tracker which provides electron identification in the region $|\eta | < 2.0$. Starting in Run 2, a new inner pixel layer, the Insertable B-Layer (IBL) [48, 49], has been inserted at a mean sensor radius of 3.3 cm. The inner detector is surrounded by a thin superconducting solenoid providing an 2 T axial magnetic field and by a lead/liquid-argon (LAr) electromagnetic calorimeter covering $|\eta | < 3.2$. A steel/scintillator-tile calorimeter provides hadronic coverage in the central pseudorapidity range ($|\eta | < 1.7$). The endcap and forward regions ($1.5< |\eta | < 4.9$) of the hadronic calorimeter are made of LAr active layers with either copper or tungsten as the absorber material. An extensive muon spectrometer with an air-core toroidal magnet system surrounds the calorimeters. Three layers of high-precision tracking chambers provide coverage in the range $|\eta | < 2.7$, while dedicated fast chambers allow triggering in the region $|\eta | < 2.4$. The ATLAS trigger system consists of a hardware-based level-1 trigger followed by a software-based high level trigger [50].

## Data sample

The data used in this analysis were collected by the ATLAS detector in *pp* collisions at $\sqrt{s} = 13$ $\text {TeV}$ at the LHC using a minimum proton bunch crossing interval of 25 ns during 2015 and 2016. In this dataset the mean number of *pp* interactions per proton bunch crossing is about 23. Events were recorded using a four-jet trigger with transverse momentum ($p_{\text {T}}$) thresholds of 100 GeV for each jet at the high-level trigger, which is fully efficient after the analysis selection requirements are applied. After requiring quality criteria for the beam, the data and the detector condition, the available dataset corresponds to an integrated luminosity of 36.7 fb$^{-1}$ with an uncertainty of $\pm 2.1\%$ for the 2015 data and $\pm 3.4\%$ for the 2016 data. The uncertainty in the integrated luminosity is obtained from a calibration of the luminosity scale using a pair of beam-separation scans performed in August 2015 and June 2016, following a methodology similar to that detailed in Ref. [51].

## Simulated samples

The dominant background from SM multijet production is estimated with a data-driven technique, while Monte Carlo (MC) simulated events are used to estimate the contribution of the $t\bar{t}$ background, to model the signals and to establish and validate the background estimation method.

The response of the detector was simulated [52] using either a GEANT4 simulation [53] or a fast parameterised simulation [54] of the calorimeter response and GEANT4 for everything else. To account for additional *pp* interactions in the same and nearby bunch crossings (pile-up), a set of minimum-bias interactions was generated using Pythia 8.186 [55] with the A2 set of parameters (tune) [56] and the MSTW2008LO [57, 58] parton distribution function (PDF) set and was superimposed on the hard scattering events. The EvtGen v1.2.0 program [59] was used to simulate properties of bottom and charm hadron decays for all samples. Corrections were applied to the simulated events to account for differences between data and simulation for the efficiency of identifying jets originating from the fragmentation of *b*-quarks, together with the probability for mistagging light-flavour and charm-quark jets.

Background samples of multijet production were simulated with $2\rightarrow 2$ matrix elements (ME) at leading order (LO) using the Pythia 8.186 event generator. The renormalisation and factorisation scales were set to the average $p_{\text {T}}$ of the two leading jets. The ATLAS A14 tune [60] of parton shower and multiple parton interaction parameters was used together with the NNPDF23LO PDF set [61].

Top-pair production events were simulated using the Powheg-Box v2 [62] generator with the CT10 PDF set. The top mass was set to 172.5 $\text {GeV}$. The $h_{\mathrm {damp}}$ parameter, which regulates the transverse momentum of the first extra gluon emission beyond the Born configuration (and thus controls the transverse momentum of the $t\bar{t}$ system), was set to the mass of the top quark. The parton shower, hadronisation, and underlying event were simulated using Pythia 6.428 [63] with the CTEQ6L1 PDF set and the corresponding Perugia 2012 tune (P2012) [64]. The sample was normalised using the next-to-next-to-leading-order (NNLO) cross-section including the resummation of soft gluon emission at next-to-next-to-logarithmic (NNLL) accuracy using Top++2.0 [65].

The search considers three benchmark signals: the pair production of top squarks, colorons and sgluons with decays into two jets for each resonance.

Signal samples were generated using MG5_aMC@NLO [66] v2.2.3 interfaced to Pythia 8.186 with the A14 tune for the modelling of the parton shower, hadronisation and underlying event. The ME calculation was performed at leading order and, for the top squark signal, includes the emission of up to two additional partons. The merging with the parton shower was done using the CKKW-L [67] prescription, with a merging scale set to one quarter of the pair-produced resonance mass. The PDF set used for the generation is NNPDF23LO. For the top squark signal generation all the non-SM particle masses were set to 5 $\text {TeV}$ except for the top squark mass ($m_{\tilde{t}}$) itself. The top squark was decayed in Pythia 8 assuming a 100% branching ratio into $\bar{b}\bar{s}$. Its width is expected to be small, and negligible with respect to the detector resolution. This set of samples is also used to interpret the analysis for the case where both top squarks decay into light quarks, since the analysis is not sensitive to the flavour content of the jets. The top squark pair-production cross-sections were calculated at next-to-leading order (NLO) in the strong coupling constant, adding the resummation of soft gluon emission at next-to-leading-logarithmic accuracy [68–70]. The nominal cross-section and its uncertainty were taken from an envelope of cross-section predictions using different PDF sets and factorisation and renormalisation scales, as described in Ref. [71]. The coloron samples were generated with the model described in Ref. [72], where the couplings of the vector colour octet to all particles except light quarks were set to zero. The LO cross-sections from the event generator were used. The coloron samples are also used to interpret the result in the context of sgluon pair-production, where they are scaled to the sgluon cross-section computed at NLO with MG5_aMC@NLO [73, 74]. The sgluons are assumed to decay into two gluons, which in this analysis are not distinguished from quark-initiated jets.

## Event reconstruction

Candidate jets are reconstructed from three-dimensional topological energy clusters [75] in the calorimeter using the anti-$k_t$ jet algorithm [76], as implemented in the FastJet package [77], with a radius parameter of 0.4. Each topological cluster is calibrated to the electromagnetic energy scale prior to jet reconstruction. The reconstructed jets are then calibrated to the particle level by the application of a jet energy scale (JES) calibration derived from simulation and in situ corrections based on 13 $\text {TeV}$ data [78–80]. The TightBad cleaning quality criteria [81] are imposed to identify jets arising from non-collision sources or detector noise. Any event containing at least one jet failing quality requirements with $p_{\text {T}} {}>20$ $\text {GeV}$ is removed.

Jets containing *b*-hadrons (*b*-jets) are tagged by a multivariate algorithm (MV2c10) using information about the impact parameters of inner detector tracks associated with the jet, the presence of displaced secondary vertices, and the reconstructed flight paths of *b*- and *c*-hadrons inside the jet [82]. A working point with a 77% efficiency, as determined in a simulated sample of $t\bar{t}$ events, is chosen. The corresponding rejection factors against simulated jets originating from *c*-quarks and from light quarks or gluons are 4.5 and 130, respectively [83].

## Event selection

Each event is required to have a reconstructed primary vertex with at least two associated tracks with $p_{\text {T}} > 400$ $\text {MeV}$ and a position consistent with the beamspot envelope. If more than one such vertex is found, the vertex with the largest $\sum p_{\text {T}} ^2$ of the associated tracks is chosen.

The final state under consideration consists of four jets forming two pairs originating from a pair of equal-mass resonances. After the trigger requirement, only events with at least four reconstructed jets with $p_{\text {T}} > 120$ $\text {GeV}$ and $|\eta | < 2.4$ are retained in the analysis.

The analysis strategy exploits the case where the resonances are produced with a significant transverse momentum. As a result the decay products are expected to be close to each other. Taking advantage of this property, candidate resonances are constructed by pairing the four leading jets in the event. Two jet pairs are identified by the following quantity:

$$\begin{aligned} \Delta R_{\text {min}} = \text {min}\left\{ \sum _{i=1,2} |\Delta R_{i} - 1| \right\} , \end{aligned}$$

where $\Delta R_{i}$ is the angular distance between the two jets for the $i{\mathrm {th}}$ pair and the sum is over the two pairs of dijets. The offset of $-1$ is chosen to maximise the signal efficiency for the masses of interest while minimising the effects of soft jets from radiated gluons being recombined with their parent jets in multijet topologies.

The above criteria define the analysis preselection. Additional requirements are applied to further enhance the signal purity. These are based on four discriminating variables established from simulation studies and previous ATLAS searches [38, 43, 44].

To reduce the non-resonant multijet background, for which the pairing efficiency is expected to be poor, a quality criterion is applied to the pairing metric. Resonances with higher masses are produced with a lower boost, and their decay products are less collimated. To compensate for the larger (smaller) angular separation between the jets at high (low) mass this requirement is made dependent on the average reconstructed mass of the two resonance candidates in the event, $m_{\text {avg}}$. The event is discarded if the best combination of the four leading jets satisfies:

$$\begin{aligned} \Delta R_{\mathrm {min}}&> -0.002 \cdot (m_{\mathrm {avg}}/\text {GeV}-225) + 0.72 \quad \mathrm {if}\; m_{\mathrm {avg}} \le 225~\text {GeV}, \\ \Delta R_{\mathrm {min}}&> +0.0013 \cdot (m_{\mathrm {avg}}/\text {GeV}-225) + 0.72 \quad \mathrm {if}\; m_{\mathrm {avg}} > 225~\text {GeV}. \end{aligned}$$

After boosting the system formed by the two resonances into its centre-of-mass frame, the magnitude of the cosine of the angle that either of them forms with the beamline is denoted as $|\cos (\theta ^*)|$. Background jets from multijet production frequently originate from *t*-channel gluon exchange and are preferentially produced in the forward region, with $|\cos (\theta ^*)|$ close to one. Jets originating from the signal are instead expected to be more central and lead to small $|\cos (\theta ^*)|$ values.

Since the two reconstructed resonances are expected to have equal mass, their mass difference is a powerful discriminant between signal and background. The mass asymmetry ($\mathcal {A}$) is defined as:

$$\begin{aligned} \mathcal {A} = \frac{|m_{1} - m_{2}|}{m_{1} + m_{2}}, \end{aligned}$$

where $m_{1}$ and $m_{2}$ are the invariant masses of the two reconstructed dijet pairs. The value of $\mathcal {A}$ is expected to peak at zero for well-paired signal events and to have larger values for background events

**Fig. 2:**
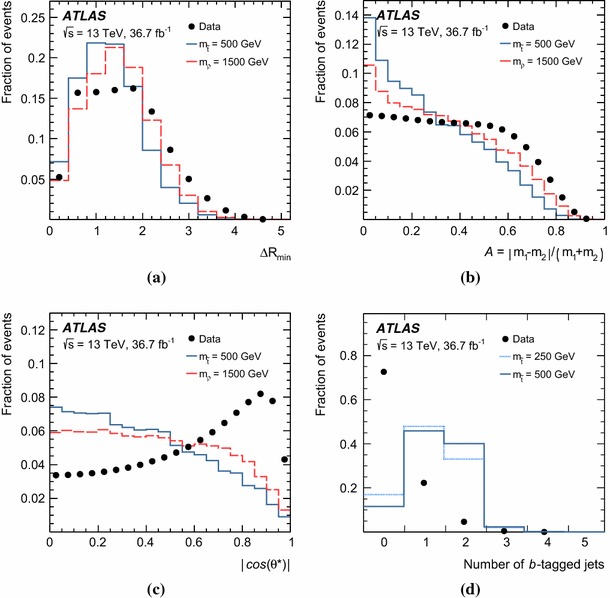
The distributions of the (**a**) smallest angular separation between the two jets in a pair ($\Delta R_{\text {min}}$), the (**b**) mass asymmetry ($\mathcal {A}$), the (**c**) pair production angle $|\cos (\theta ^*)|$ and the (**d**) multiplicity of *b*-tagged jets. The observed data (black dots) are compared with the distributions expected from a top squark with a mass of 250 $\text {GeV}$ (solid blue line) or 500 $\text {GeV}$ (azure dotted line) and a coloron with a mass of 1500 $\text {GeV}$ (red dashed line). The distributions are normalised to unity and shown at preselection, after the requirement of four jets paired into two candidate resonances

The distributions of $\Delta R_{\mathrm {min}}$, $\mathcal {A}$ and $|\cos (\theta ^*)|$ after preselection are shown for data, a top squark sample with a mass of $m_{\tilde{t}}=500$ $\text {GeV}$ and a coloron sample with mass $m_{\rho }=1500$ $\text {GeV}$ in Fig. 2a–c. Because of the very small expected signal purity (below 2%) before additional selection criteria are applied the data distributions can be viewed as representative of the expected background. Two additional requirements, $\mathcal {A} < 0.05$ and $|\cos (\theta ^*)|<0.3$, define the inclusive signal region (SR) selection, targeting resonance decays into light quark or gluon jets. The selections are determined in an optimisation procedure that maximises the expected signal significance.

When the dominant RPV couplings involve third-generation quarks ($\lambda ^{''}_{3i3}$), a *b*-quark is expected from each of the top squark decays. A dedicated *b*-tagged SR selection is used for this scenario. On top of the requirements applied in the inclusive selection it requires at least two *b*-tagged jets to be present in the event, which significantly reduces the multijet background. The distribution of the number of *b*-tagged jets after pairing the four jets into candidate resonances is shown for data and two top squark signals with masses of 250 and 500 $\text {GeV}$ in Fig. 2d. An additional factor of about two in background reduction is gained by requiring each of the two *b*-jets to be associated with a different reconstructed resonance. This is particularly effective in reducing the contribution of $g\rightarrow b\bar{b}$ splittings, where the two *b*-jets are typically very collimated.

The final analysis discriminant is the average mass of the two reconstructed resonances:

$$\begin{aligned} m_{\mathrm {avg}}=\frac{1}{2}(m_1+m_2). \end{aligned}$$

**Fig. 3:**
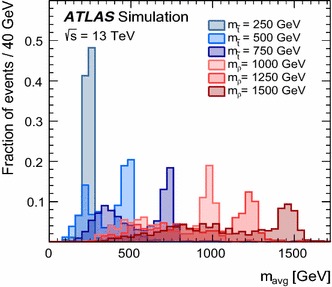
Distribution of the average mass, $m_{\text {avg}}$, in the inclusive signal region for simulated top squark signals with $m_{\tilde{t}}=250$, 500, and 750 and coloron signals with $m_{\rho }=1000$, 1250, and 1500 $\text {GeV}$

A peak in $m_{\text {avg}}$ at a mass of about that of the resonance is expected for the signal, over a non-peaking background from multijet processes. Figure 3 shows the expected $m_{\text {avg}}$ distribution for signal samples with different masses. For each mass hypothesis a counting experiment is performed in a window of the $m_{\text {avg}}$ variable optimised to maximise the expected signal significance. The windows range from a 10 $\text {GeV}$ width for a 100 $\text {GeV}$ top squark to a 200 $\text {GeV}$ width for a 1500 $\text {GeV}$ coloron. The mass window for the highest target mass considered, of 2000 $\text {GeV}$, has no upper edge. When the mass difference between two signal samples is smaller than the experimental resolution, their selected mass windows partially overlap.

**Fig. 4:**
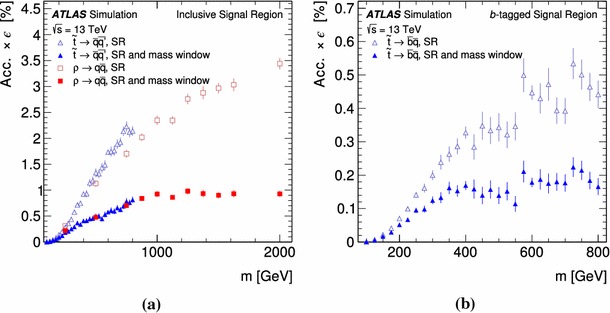
The acceptance times efficiency (Acc.$\times \epsilon$) of the **a** inclusive and **b** *b*-tagged signal region selection as a function of the resonance mass, *m*, before and after the mass window requirements are applied. Top squark signals are indicated by the blue triangles, coloron by the red squares. The statistical uncertainties are indicated by vertical bars

**Table 1:** MC predictions of the number of signal events corresponding to 36.7 fb$^{-1}$ of data after applying each of the event selection requirements, except for the mass window. Top squark masses of $m_{\tilde{t}}=100$ $\text {GeV}$ and $m_{\tilde{t}}=500$ $\text {GeV}$, and a coloron mass of 1500 $\text {GeV}$ are shown. The statistical uncertainty of the MC simulation is shown for each selection

Selection	$m_{\tilde{t}}=100$ $\text {GeV}$	$m_{\tilde{t}}=500$ $\text {GeV}$	$m_{\rho }=1500$ $\text {GeV}$
Total	$(558.0\pm 0.6)\times 10^{5}$	$19000\pm 130$	$1710\pm 10$
Trigger	$221900\pm 420$	$11900\pm 100$	$1650\pm 10$
$\Delta R_{\mathrm {min}}$	$18910\pm 120$	$2470\pm 50$	$1050\pm 5$
Inclusive selection	$1359\pm 36$	$253\pm 16$	$51\pm 2$
*b*-tagged selection	$569\pm 24$	$65\pm 8$	–

The MC predictions of signal event yields in 36.7 fb$^{-1}$ of data are shown in Table 1 after each different requirement of the event selection is applied. The acceptance times efficiency of the inclusive and *b*-tagged signal region selections as a function of the signal mass are shown before and after applying the $m_{\text {avg}}$ mass window requirement in Fig. 4. The acceptance of the signal region selections increases for large masses due to the four jets from the signal having a larger $p_{\text {T}}$. However, as the jet pairing does not always correctly assign the resonance candidates for high masses, the signal has a tail extending to low $m_{\text {avg}}$ values, degrading the efficiency of the mass window selection.

## Background estimation

The dominant background from multijet production is estimated directly from data, with a method that predicts both the normalisation and the shape of the $m_{\text {avg}}$ distribution. In the *b*-tagged selection, for $m_{\text {avg}}$ below 200 $\text {GeV}$, the $t\bar{t}$ contribution becomes significant, and is estimated from simulation.

**Fig. 5:**
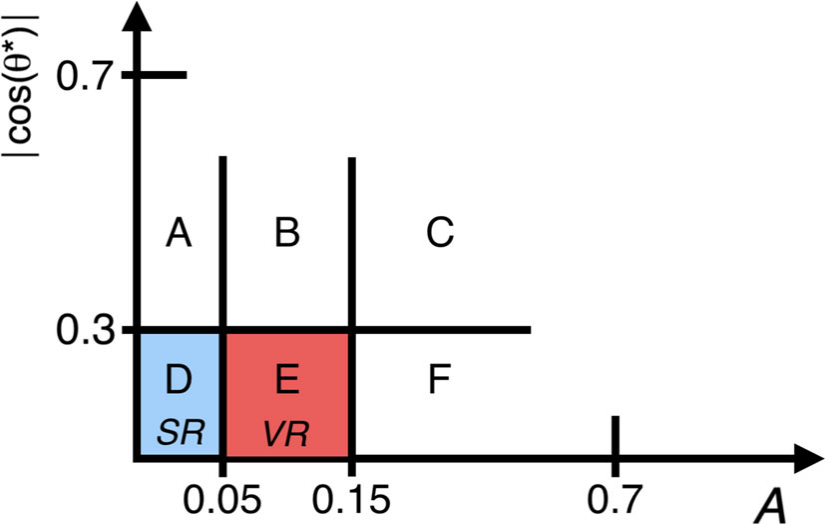
Definition of the control and validation regions in the $\mathcal {A}$ and $|\cos (\theta ^*)|$ plane used to estimate the multijet background

For the inclusive selection, the $m_{\text {avg}}$ distribution for the background is obtained from data. For each $m_{\text {avg}}$ bin the data sample is divided into four regions: one region where the signal region selection is applied (D) and three background-dominated control regions (A, C and F). The variables used to define the different regions, summarised in Fig. 5, are $\mathcal {A}$ and $|\cos (\theta ^*)|$. Provided the two variables defining the regions are uncorrelated, and signal leakage in the background-dominated regions can be neglected, the amount of background in the region of interest D can be predicted from the observed numbers of events in the control regions as $N_\mathrm {D} = N_\mathrm {A} \times N_\mathrm {F}/N_\mathrm {C}$. The linear correlation between the $|\cos (\theta ^*)|$ and $\mathcal {A}$ variables is evaluated in data and simulated multijet samples, where it amounts to 1.8 and 2.2%, respectively. Significant correlations are observed in data at large $m_{\text {avg}}$ and high $\mathcal {A}$ values; to reduce their impact on the background estimate the $\mathcal {A}$–$|\cos (\theta ^*)|$ plane is restricted to $0.0<\mathcal {A}<0.7$ and $0.0<|\cos (\theta ^*)|<0.7$. Two additional regions (B and E) are defined in the $\mathcal {A}$–$|\cos (\theta ^*)|$ plane. The validation region (VR), region E, is used to test the performance of the data-driven method and assign an uncertainty to the background estimate. The validation region is defined with the same selections as for the signal region, but with the asymmetry requirement changed from $\mathcal {A} < 0.05$ to $0.05< \mathcal {A} < 0.15$. The background contribution in the VR is estimated by $N_\mathrm {E} = N_\mathrm {B} \times N_\mathrm {F}/N_\mathrm {C}$. In the inclusive selection the data-driven estimate also accounts for the contribution from the $t\bar{t}$ production, which amounts to less than 1% of the total background for $m_{\mathrm {avg}}<200$ $\text {GeV}$, and is negligible above.

For the *b*-tagged selection, where the background is relatively small, the signal contamination in region $\mathrm {A}$ can be significant and potentially bias the result of the background estimate. The multijet background for this selection is thus estimated in two steps. The shape of the $m_{\text {avg}}$ distribution is first predicted in a region with a *b*-tag veto (zero-tag) and then extrapolated to the *b*-tagged signal region. The $m_{\text {avg}}$ distribution in the zero-tag region is obtained with a data-driven estimate, analogously to the inclusive selection. The zero-tag prediction is then extrapolated to the *b*-tagged selection by means of projection factors computed bin by bin in $m_{\text {avg}}$, similarly to the approach described in Ref. [44]. The projection factors, for a given $m_{\text {avg}}$ bin and region in the $\mathcal {A}$–$|\cos (\theta ^*)|$ plane, are defined as the ratio of the numbers of events with two *b*-tags and zero *b*-tags, $N_{\mathrm {two-}b\mathrm {-tags}}/N_{\mathrm {zero-}b\mathrm {-tags}}$, within that region. The method assumes the projection factors to be constant across the $\mathcal {A}$–$|\cos (\theta ^*)|$ plane. They are evaluated in region F, where a negligible signal contamination is expected. The contributions from multi-jet and $t\bar{t}$ production scale differently between the zero- and *b*-tagged selection. Hence, simulated samples are used to subtract the $t\bar{t}$ contribution in all control regions. The $t\bar{t}$ estimate in the signal region is then obtained directly from the simulation, considering all relevant modelling and experimental uncertainties.

**Table 2:** Observed numbers of events and the predicted $t\bar{t}$ contributions in each of the regions used in the background estimate, for each *b*-tag multiplicity. The expected fractional signal contributions are shown for the mass windows corresponding to $m_{\tilde{t}}=125$, 250, 500, and 800 $\text {GeV}$. For the $m_{\tilde{t}}=125$ and 250 $\text {GeV}$ mass windows the fractions of $t\bar{t}$ are also shown. The $t\bar{t}$ systematic uncertainties include both the detector-level uncertainties and the theoretical uncertainties, as described in Sect. 8

Target mass			$125~\text {GeV}$		$250~\text {GeV}$		$500~\text {GeV}$	$800~\text {GeV}$
Region	$N_{\mathrm {Data}}$	$N_{t\bar{t}}$ (± stat. ± syst.)	$[120, 135]~\text {GeV}$		$[230, 260]~\text {GeV}$		$[455, 515]~\text {GeV}$	$[720, 820]~\text {GeV}$
			$\frac{N_{\mathrm {Sig}}}{N_{\mathrm {Data}}}$ (%)	$\frac{N_{t\bar{t}}}{N_{\mathrm {Data}}}$ (%)	$\frac{N_{\mathrm {Sig}}}{N_{\mathrm {Data}}}$ (%)	$\frac{N_{t\bar{t}}}{N_{\mathrm {Data}}}$ (%)	$\frac{N_{\mathrm {Sig}}}{N_{\mathrm {Data}}}$ (%)	$\frac{N_{\mathrm {Sig}}}{N_{\mathrm {Data}}}$ (%)
Inclusive selection								
A	256,937	$5044 \pm 76 \pm 1092$	7.2	5.8	5.6	0.28	3.1	1.7
B	508,589	$8900 \pm 100 \pm 1410$	1.95	4.7	1.3	0.24	0.6	0.4
C	1,154,721	$13{,}080 \pm 120 \pm 1950$	0.17	2.3	0.16	0.43	0.07	0.07
D (SR)	154,750	$3826 \pm 66 \pm 812$	14.0	7.0	10.5	0.31	6.3	3.5
E (VR)	307,268	$6578 \pm 87 \pm 995$	3.86	6.0	2.2	0.33	1.4	0.8
F	694,492	$9920 \pm 110 \pm 1900$	0.29	3.5	0.3	0.57	0.2	0.13
Zero *b*-tags selection								
A	184,432	$580 \pm 27 \pm 85$	0.56	0.53	0.44	0.11	0.50	0.46
B	366,003	$1165 \pm 38 \pm 213$	0.14	0.57	0.18	0.10	0.12	0.19
C	834,944	$2352 \pm 53 \pm 399$	0.07	0.66	0.03	0.13	0.02	0.04
D	110,071	$506 \pm 26 \pm 94$	1.18	0.72	1.65	0.16	1.48	1.3
E	219,366	$831 \pm 32 \pm 183$	0.45	0.67	0.24	0.11	0.10	0.4
F	498,751	$1743 \pm 46 \pm 291$	0.07	0.83	0.08	0.20	0.08	0.04
*b*-tagged selection								
A	8484	$2375 \pm 53 \pm 902$	82	64	112	0.94	43	18.2
B	16,113	$3614 \pm 64 \pm 867$	23	53	23	1.4	11	4.5
C	32,759	$3681 \pm 63 \pm 840$	1.2	31	1.3	2.2	0.38	0.1
D (SR)	5603	$1707 \pm 44 \pm 499$	135	64	181	0.54	70	24.6
E (VR)	10,531	$2678 \pm 55 \pm 499$	38	58	35	0.9	20	9.2
F	20,856	$2904 \pm 56 \pm 721$	2.3	37	3.1	2.7	1.4	0.7

**Fig. 6:**
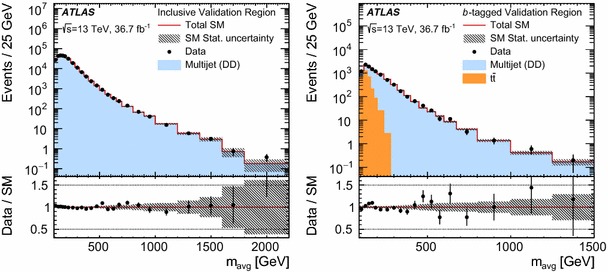
The $m_{\text {avg}}$ spectrum in the inclusive (left) and *b*-tagged (right) validation regions. The data (black points) are compared to the total background prediction (red line) estimated with the data-driven method. The fraction of background coming from top-pair production is shown in orange. The statistical uncertainties of the prediction are shown by the grey hatched band

**Fig. 7:**
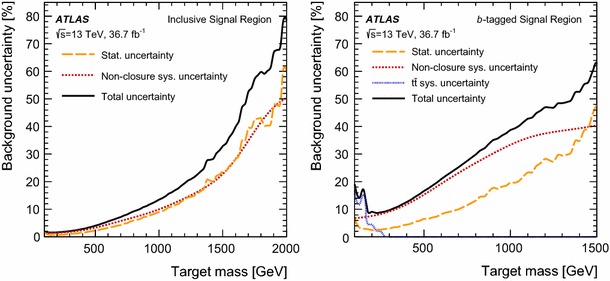
The uncertainty in the data-driven background estimate in the inclusive (left) and *b*-tagged (right) signal regions, computed in the $m_{\text {avg}}$ mass windows defined for different target masses. The uncertainty arising from the non-closure in the validation region is shown with a red short-dashed line and compared with the statistical uncertainty of the data-driven prediction shown as an orange dashed line. The additional uncertainty in the MC estimate of the top background in the *b*-tagged signal region is shown as a dotted blue line. The total uncertainty, obtained by adding in quadrature the different components, is indicated by a black solid line

The observed number of events in each of the regions used in the background estimate before the mass window requirements are applied, together with the expected signal contamination in few representative mass windows, are shown for both the inclusive and *b*-tagged selections in Table 2. The $m_{\text {avg}}$ distribution in the validation region for the inclusive and *b*-tagged signal regions is shown in Fig. 6. Within the statistical uncertainties the method reproduces both the normalisation and the shape of $m_{\text {avg}}$ in the VRs. The level of agreement observed in the VRs is used to derive a systematic uncertainty in the background estimate in the SR. In each $m_{\text {avg}}$ mass window the difference between the observed data and the estimation in the VR (non-closure) is computed. The larger of the observed non-closure in the VR and the statistical uncertainty of the data-driven method is assigned as an uncertainty in the background estimates. To reduce the effect of statistical fluctuations in the non-closure and avoid quoting an unphysically small value of the systematic uncertainty for the mass windows where it changes sign, this uncertainty is further smoothed as a function of $m_{\text {avg}}$. The Nadaraya–Watson [84, 85] kernel regression estimate is used for the smoothing, with a bandwidth of 500 $\text {GeV}$ (meaning that the quartiles of the kernels are placed at $\pm 125$ $\text {GeV}$). The uncertainties assigned to the background estimate in the inclusive and *b*-tagged signal regions are summarised in Fig. 7.

## Systematic uncertainties

While the multijet background uncertainties pertain primarily to the estimation method itself, the top background and the signals are also affected by uncertainties related to the description of detector effects and to the physics modelling of the MC simulation.

The dominant detector-related systematic effects are due to the uncertainties in the jet energy scale [80] and resolution [86] and from the *b*-tagging efficiency and mistag rate [83].

Since MC simulation is used to determine the contribution from top events in the *b*-tagged signal region, systematic uncertainties related to the choice of MC generator for the process need to be estimated. These are evaluated by comparing the nominal samples to additional samples with systematic variations. A modelling uncertainty is derived by comparing the predictions of the nominal sample with a sample produced with Powheg interfaced with Herwig++ 2.7.1, or with MG5_aMC@NLO interfaced with Herwig++. In addition, the difference in the prediction obtained by modifying the parton-shower intensity and the $h_{\mathrm {damp}}$ parameter in the nominal sample is taken as an uncertainty. The $t\bar{t}$ systematic uncertainty on the total background is as large as 20% for $m_{\text {avg}}$ below 200 $\text {GeV}$, becoming negligible above 200 $\text {GeV}$.

The total detector-related uncertainties in the signal are about 10% for the inclusive SR and about 15% for the two-*b*-tagged SR. For top squark production the nominal signal cross-sections and their uncertainties are taken from an envelope of cross-section predictions derived using different PDF sets and different factorisation and renormalisation scales, as described in Ref. [71]. The theoretical uncertainties in the acceptance of the signal simulation include variations of the renormalisation and factorisation scales, the CKKW-L merging scales, and the value of the strong coupling constant in MG5_aMC@NLO as well as parton shower uncertainties in Pythia 8 evaluated from variations of the A14 parameter set. After normalising the samples using the same cross-section, the difference between the yields from the nominal and varied samples in the mass window, which is typically below 1%, is considered as an uncertainty.

## Results and interpretation

**Fig. 8:**
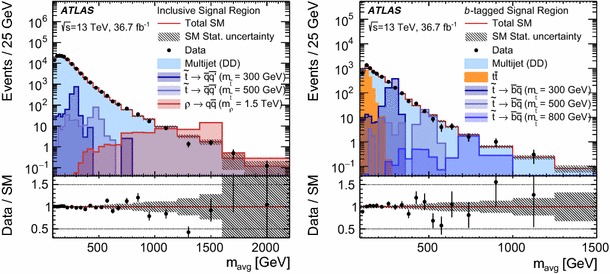
The $m_{\text {avg}}$ spectrum in the inclusive (left) and *b*-tagged (right) signal regions. The data (black points) are compared to the total background prediction (red line) estimated with the data-driven method. The fraction of background coming from top-pair production is shown in orange. The statistical uncertainties of the prediction are shown by the grey hatched band. Signals of different masses are overlaid in different colours

The $m_{\text {avg}}$ distributions in the inclusive and *b*-tagged signal regions are shown in Fig. 8. Agreement is observed between data and the expected background. The expected numbers of background and signal events in the SR and their uncertainties are reported for the mass windows defined for top squark and coloron signals in Tables 3 and 4, respectively, together with the observed events in data. Table 5 presents the numbers in the top squark mass windows of the two-*b*-tagged signal region.

To estimate the compatibility of the data with a generic resonance mass hypothesis, the $m_{\text {avg}}$ distribution is scanned in 12.5 $\text {GeV}$ steps. The $m_{\text {avg}}$ window for an arbitrary mass is obtained from a linear fit to the lower and upper edges of the windows obtained for the simulated signal masses. For each mass a background $p_0$-value is computed for the inclusive and *b*-tagged signal regions. The largest deviation is found in the *b*-tagged signal region for a mass of 463 $\text {GeV}$, corresponding to a local $p_0$ value of 0.05.

The expected $p_0$ values in each mass window are also evaluated for potential signals. At least three-standard-deviation $(3\sigma )$ signal sensitivity is expected for top squark masses up to 350 $\text {GeV}$ with the inclusive signal region and 450 $\text {GeV}$ with the *b*-tagged signal region. For colorons a greater than $3\sigma$ sensitivity is expected for masses up to 1400 $\text {GeV}$.

**Table 3:** Observed numbers of events in the data, $N_{\mathrm {Data}}$, the estimated numbers of background events, $N_{\mathrm {Bkg}}$, and the expected numbers of top squark signal events, $N_{\mathrm {Sig}}$, in the top squark mass windows of the inclusive signal region. Separate statistical and systematic uncertainties are given

$m_{\tilde{t}}$ [GeV]	Window [$\text {GeV}$]	$N_{\mathrm {Data}}$	$N_{\mathrm {Bkg}}$ (± stat. ± syst.)	$N_{\mathrm {Sig}}$ (± stat. ± syst.)
100	[100, 110]	5899	$5910 \pm 90 \pm 70$	$519 \pm 23 \pm 68$
125	[120, 135]	13497	$13450 \pm 120 \pm 180$	$1890 \pm 50 \pm 190$
150	[140, 160]	18609	$18390 \pm 130 \pm 250$	$2540 \pm 50 \pm 130$
175	[165, 185]	17742	$17800 \pm 130 \pm 250$	$2280 \pm 50 \pm 210$
200	[185, 210]	19844	$19660 \pm 140 \pm 290$	$2250 \pm 50 \pm 170$
225	[210, 235]	14898	$15180 \pm 120 \pm 230$	$1620 \pm 40 \pm 100$
250	[230, 260]	13689	$13750 \pm 110 \pm 220$	$1440 \pm 80 \pm 140$
275	[255, 285]	9808	$9860 \pm 100 \pm 170$	$1010 \pm 70 \pm 80$
300	[275, 310]	8514	$8790 \pm 90 \pm 160$	$789 \pm 52 \pm 31$
325	[300, 335]	6180	$6330 \pm 80 \pm 120$	$600 \pm 50 \pm 50$
350	[320, 365]	5802	$5900 \pm 70 \pm 120$	$509 \pm 39 \pm 19$
375	[345, 390]	4113	$4250 \pm 60 \pm 90$	$324 \pm 25 \pm 31$
400	[365, 415]	3531	$3590 \pm 60 \pm 90$	$274 \pm 14 \pm 18$
425	[385, 440]	3108	$3010 \pm 50 \pm 80$	$198 \pm 23 \pm 10$
450	[410, 465]	2281	$2230 \pm 40 \pm 60$	$154 \pm 17 \pm 27$
475	[430, 490]	1906	$1920 \pm 40 \pm 60$	$116 \pm 12 \pm 8$
500	[455, 515]	1495	$1513 \pm 35 \pm 49$	$94 \pm 10 \pm 8$
525	[475, 540]	1318	$1327 \pm 33 \pm 46$	$71 \pm 7 \pm 4$
550	[500, 565]	1050	$1048 \pm 29 \pm 39$	$48.5 \pm 5.4 \pm 2.2$
575	[520, 590]	924	$912 \pm 27 \pm 36$	$44 \pm 4 \pm 4$
600	[545, 620]	745	$744 \pm 25 \pm 31$	$36.9 \pm 1.6 \pm 2.3$
625	[565, 645]	645	$626 \pm 22 \pm 28$	$30.3 \pm 2.8 \pm 3.4$
650	[585, 670]	536	$554 \pm 21 \pm 26$	$23.3 \pm 2.1 \pm 1.9$
675	[610, 695]	438	$473 \pm 19 \pm 24$	$20.3 \pm 1.6 \pm 0.9$
700	[630, 720]	404	$422 \pm 18 \pm 22$	$15.4 \pm 1.2 \pm 0.9$
725	[655, 745]	341	$335 \pm 16 \pm 18$	$13.6 \pm 1.0 \pm 0.9$
750	[675, 770]	306	$310 \pm 16 \pm 18$	$12.4 \pm 0.9 \pm 0.9$
775	[700, 795]	265	$243 \pm 14 \pm 14$	$9.7 \pm 0.7 \pm 0.7$
800	[720, 820]	238	$205 \pm 12 \pm 13$	$8.5 \pm 0.6 \pm 0.6$

**Table 4:** Observed numbers of events in the data, $N_{\mathrm {Data}}$, the estimated numbers of background events, $N_{\mathrm {Bkg}}$, and the expected numbers of coloron signal events, $N_{\mathrm {Sig}}$, in the coloron mass windows of the inclusive signal region. Separate statistical and systematic uncertainties are given

$m_{\rho }$ [GeV]	Window [$\text {GeV}$]	$N_{\mathrm {Data}}$	$N_{\mathrm {Bkg}}$ (± stat. ± syst.)	$N_{\mathrm {Sig}}$ (± stat. ± syst.)
500	[455, 515]	1495	$1513 \pm 35 \pm 15$	$23000 \pm 1900 \pm 1200$
625	[565, 645]	645	$626 \pm 22 \pm 35$	$7050 \pm 370 \pm 350$
750	[675, 770]	306	$310 \pm 15 \pm 30$	$2510 \pm 170 \pm 120$
875	[790, 900]	166	$144 \pm 10 \pm 16$	$1020 \pm 56 \pm 23$
1000	[900, 1025]	79	$96 \pm 9 \pm 8$	$416 \pm 25 \pm 17$
1125	[1010, 1155]	46	$58 \pm 7 \pm 5$	$154 \pm 8 \pm 5$
1250	[1120, 1280]	27	$36 \pm 5 \pm 3$	$73 \pm 4 \pm 4$
1375	[1235, 1410]	9	$17 \pm 3 \pm 3$	$51.0 \pm 2.0 \pm 1.2$
1500	[1345, 1535]	13	$14 \pm 3 \pm 1.6$	$12.9 \pm 0.8 \pm 0.4$
1625	[1455, 1665]	7	$8.70 \pm 2.56 \pm 0.6$	$12.9 \pm 0.8 \pm 0.4$
1750	[1565, 1790]	6	$4.79 \pm 2.04 \pm 2.55$	$2.80 \pm 0.12 \pm 0.13$
1875	[1680, 1920]	4	$5.27 \pm 2.15 \pm 3.51$	$1.33 \pm 0.07 \pm 0.07$
2000	[1790, $\infty$]	2	$2.07 \pm 1.24 \pm 0.4$	$0.64 \pm 0.06 \pm 0.06$

**Table 5:** Observed numbers of events in the data, $N_{\mathrm {Data}}$, the estimated numbers of background events, $N_{\mathrm {Bkg}}$, and the expected numbers of top squark signal events, $N_{\mathrm {Sig}}$, in the top squark mass windows of the *b*-tagged signal region. Separate statistical and systematic uncertainties are given

$m_{\tilde{t}}$ [$\text {GeV}$]	Window [$\text {GeV}$]	$N_{\mathrm {Data}}$	$N_{\mathrm {Bkg}}$ (± stat. ± syst.)	$N_{\mathrm {Sig}}$ (± stat. ± syst.)
100	[100, 110]	256	$285 \pm 18 \pm 51$	$308 \pm 18 \pm 52$
125	[120, 135]	803	$798 \pm 28 \pm 107$	$1090 \pm 40 \pm 140$
150	[140, 160]	809	$789 \pm 23 \pm 132$	$1510 \pm 40 \pm 130$
175	[165, 185]	544	$555 \pm 16 \pm 47$	$1300 \pm 40 \pm 140$
200	[185, 210]	592	$554 \pm 13 \pm 47$	$1220 \pm 40 \pm 110$
225	[210, 235]	414	$436 \pm 11 \pm 35$	$893 \pm 28 \pm 90$
250	[230, 260]	416	$385 \pm 10 \pm 32$	$750 \pm 60 \pm 120$
275	[255, 285]	302	$283 \pm 8 \pm 24$	$480 \pm 50 \pm 60$
300	[275, 310]	242	$250 \pm 8 \pm 23$	$390 \pm 40 \pm 50$
325	[300, 335]	181	$179 \pm 6 \pm 17$	$273 \pm 33 \pm 34$
350	[320, 365]	169	$161 \pm 6 \pm 16$	$225 \pm 25 \pm 20$
375	[345, 390]	110	$111 \pm 5 \pm 12$	$147 \pm 16 \pm 22$
400	[365, 415]	80	$96 \pm 4 \pm 11$	$114 \pm 9 \pm 12$
425	[385, 440]	85	$79 \pm 4 \pm 10$	$76 \pm 14 \pm 11$
450	[410, 465]	71	$54.2 \pm 3.0 \pm 7.1$	$48 \pm 9 \pm 10$
475	[430, 490]	67	$46.8 \pm 2.7 \pm 6.5$	$40 \pm 7 \pm 5$
500	[455, 515]	38	$35.8 \pm 2.3 \pm 5.3$	$26 \pm 5 \pm 5$
525	[475, 540]	31	$35.1 \pm 2.3 \pm 5.5$	$21.7 \pm 3.9 \pm 2.8$
550	[500, 565]	20	$30.2 \pm 2.1 \pm 5.0$	$12.4 \pm 2.5 \pm 2.3$
575	[520, 590]	14	$26.3 \pm 2.0 \pm 4.6$	$17.5 \pm 2.7 \pm 3.5$
600	[545, 620]	14	$19.5 \pm 1.6 \pm 3.5$	$11.4 \pm 0.9 \pm 1.5$
625	[565, 645]	15	$15.8 \pm 1.4 \pm 3.0$	$9.3 \pm 1.5 \pm 1.4$
650	[585, 670]	14	$14.6 \pm 1.3 \pm 2.9$	$6.9 \pm 1.2 \pm 1.1$
675	[610, 695]	13	$13.6 \pm 1.3 \pm 2.8$	$5.5 \pm 0.8 \pm 0.6$
700	[630, 720]	6	$12.1 \pm 1.2 \pm 2.6$	$4.3 \pm 0.6 \pm 0.5$
725	[655, 745]	5	$9.9 \pm 1.1 \pm 2.2$	$4.4 \pm 0.6 \pm 0.8$
750	[675, 770]	4	$8.4 \pm 0.1 \pm 1.9$	$3.4 \pm 0.5 \pm 0.5$
775	[700, 795]	8	$6.9 \pm 0.9 \pm 1.6$	$2.36 \pm 0.34 \pm 0.53$
800	[720, 820]	7	$5.3 \pm 0.7 \pm 1.3$	$1.72 \pm 0.26 \pm 0.23$

**Fig. 9:**
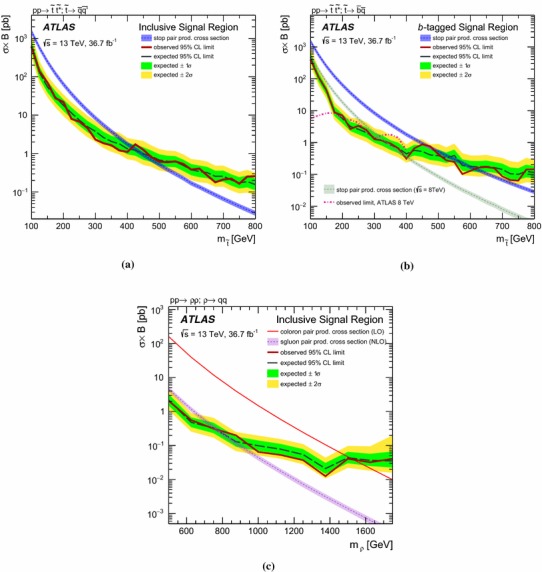
The 95% CL upper limit on the $\sigma \times B$ value compared to the theoretical cross-section for the direct pair-production of top squarks with decays into **a** $\bar{q}\bar{q'}$ or **b** $\bar{b}\bar{s}$ and **c** high-mass colorons decaying into *qq* and sgluons decaying into *gg*. The dashed black and solid red lines show the 95% CL expected and observed limits respectively, including all uncertainties except the theoretical signal cross-section uncertainty. The solid green (yellow) band around the expected limit shows the associated $\pm 1\sigma$ ($\pm 2\sigma$) ranges. The shaded coloured cross-section band indicates the $\pm 1\sigma$ variations due to theoretical uncertainties in the signal production cross-section given by renormalisation and factorisation scale and PDF uncertainties. The region of low top squark mass not shown in the plot is excluded by Refs. [41, 42]

In the absence of a statistically significant excess in data, exclusion limits are derived for the investigated signal models. The inclusive signal region is used to set a limit on top squark, sgluon and coloron production with decays into a pair of jets, while the *b*-tagged signal region is used to interpret the search for top squark pair production with decays into a *b*- and a light-quark jet. A profile likelihood ratio combining Poisson probabilities for signal and background is computed to determine the 95% CL interval for compatibility of the data with the signal-plus-background hypothesis ($\mathrm {CL}_\mathrm {s+b}$) [87]. A similar calculation is performed for the background-only hypothesis ($\mathrm {CL}_\mathrm {b}$). From the ratio of these two quantities, the confidence level for the presence of a signal ($\mathrm {CL}_\mathrm {s}$) is determined [88]. Systematic uncertainties are treated as nuisance parameters and are assumed to follow Gaussian distributions. The results are evaluated using pseudo-experiments. This procedure is implemented using a software framework for statistical data analysis, HistFitter [89]. The observed and expected 95% CL upper limits on the allowed cross-sections are shown in Fig. 9. For top squark decays into two quarks, the expected and observed mass range exclusions are between 100 and 430 $\text {GeV}$ and between 100 and 410 $\text {GeV}$, respectively. This exclusion does also apply to the pair-production of other squarks, decaying, for example, to a *d*- and a *u*-quark. If the top squark decay is into a *b*-quark and a light-quark, masses between 100 and 530 $\text {GeV}$ are expected to be excluded, with the observed exclusion ranging from 100 to 470 $\text {GeV}$ and from 480 to 610 $\text {GeV}$. Below top squark masses of about 200 $\text {GeV}$ the signal acceptance rapidly drops due to the trigger and jet requirements, and the analysis sensitivity does not surpass the 8 $\text {TeV}$ result, which was specifically optimised for low-mass signals. Pair-produced scalar gluons with decays into two gluons are excluded up to a mass of 800 $\text {GeV}$. Pair-produced colorons coupling only to light quarks are excluded up to a mass of 1500 $\text {GeV}$.

## Conclusion

A search is presented for the pair production of coloured resonances, each decaying into two jets. The analysis uses 36.7 fb$^{-1}$ of $\sqrt{s}$ = 13 TeV *pp* collision data recorded by the ATLAS experiment at the LHC in 2015 and 2016. An inclusive selection and a selection with two *b*-tagged jets in the event are defined, and counting experiments are performed in windows of the average mass of the two resonance candidates. No significant deviation from the background prediction is observed. The results are interpreted in a SUSY simplified model with a top squark as the lightest supersymmetric particle, which is pair-produced and decays promptly into two quarks through *R*-parity-violating couplings. For decays into two quarks, top squark masses in the range $100\,\text {GeV}<m_{\tilde{t}}<410\,\text {GeV}$ are excluded at 95% CL. If the top squark decays into a *b*-quark and a light quark, masses in the ranges $100\,\text {GeV}<m_{\tilde{t}}<470\,\text {GeV}$ and $480\,\text {GeV}<m_{\tilde{t}}<610\,\text {GeV}$ are excluded at 95% CL. Limits on the pair production of scalar gluons with decays into two gluons reach masses of 800 $\text {GeV}$. Vector colour-octet resonances coupling only to light quarks are excluded up to masses of 1500 $\text {GeV}$. The results improve upon previous Run 1 searches and extend the constraints on top squark masses.

## Footnotes

1 The superpartners of the left- and right-handed top quarks, $\tilde{t}_\mathrm {L}$ and $\tilde{t}_\mathrm {R}$, mix to form the two mass eigenstates $\tilde{t}_1$ and $\tilde{t}_2$, where $\tilde{t}_1$ is the lighter one. This analysis considers only the production of the $\tilde{t}_1$, which hereafter is simply referred to as $\tilde{t}$.

2 ATLAS uses a right-handed coordinate system with its origin at the nominal interaction point in the centre of the detector. The positive *x*-axis is defined by the direction from the interaction point to the centre of the LHC ring, with the positive *y*-axis pointing upwards, while the beam direction defines the *z*-axis. Cylindrical coordinates (*r*, $\phi$) are used in the transverse plane, $\phi$ being the azimuthal angle around the *z*-axis. The pseudorapidity $\eta$ is defined in terms of the polar angle $\theta$ by $\eta = - \ln \tan (\theta /2)$. Rapidity is defined as $y = 0.5\cdot \ln [(E + p_z)/(E - p_z)]$ where *E* denotes the energy and $p_z$ is the component of the momentum along the beam direction.
